# Polycomb Repressive Complex 2 in Eukaryotes—An Evolutionary Perspective

**DOI:** 10.3390/epigenomes6010003

**Published:** 2022-01-17

**Authors:** Mallika Vijayanathan, María Guadalupe Trejo-Arellano, Iva Mozgová

**Affiliations:** 1 Biology Centre, Institute of Plant Molecular Biology, Czech Academy of Sciences, 370 05 Ceske Budejovice, Czech Republic; mallika.vijayanathan@umbr.cas.cz (M.V.); maria.trejo@umbr.cas.cz (M.G.T.-A.); 2 Faculty of Science, University of South Bohemia, 370 05 Ceske Budejovice, Czech Republic

**Keywords:** polycomb, PRC2, H3K27me3, evolution, green lineage, plant, animal, fungi, algae, SAR

## Abstract

Polycomb repressive complex 2 (PRC2) represents a group of evolutionarily conserved multi-subunit complexes that repress gene transcription by introducing trimethylation of lysine 27 on histone 3 (H3K27me3). PRC2 activity is of key importance for cell identity specification and developmental phase transitions in animals and plants. The composition, biochemistry, and developmental function of PRC2 in animal and flowering plant model species are relatively well described. Recent evidence demonstrates the presence of PRC2 complexes in various eukaryotic supergroups, suggesting conservation of the complex and its function. Here, we provide an overview of the current understanding of PRC2-mediated repression in different representatives of eukaryotic supergroups with a focus on the green lineage. By comparison of PRC2 in different eukaryotes, we highlight the possible common and diverged features suggesting evolutionary implications and outline emerging questions and directions for future research of polycomb repression and its evolution.

## 1. Introduction

DNA in the eukaryotic nucleus winds around octamers of histones, forming nucleosomes, the basic units of chromatin. DNA and histones are subject to chemical modifications, such as methylation, phosphorylation, acetylation, and others, which are instructive for or correlate with chromatin structure. Remodeling of chromatin structure between more open (accessible) or more compact (inaccessible) states by chromatin-modifying and -remodeling complexes governs the distribution of DNA in the nuclear space and allows for gene activation or repression, respectively.

Among crucial modulators of chromatin structure are polycomb group (PcG) proteins, which form multi-subunit polycomb repressive complexes (PRCs) (reviewed in the work of [1,2,3,4,5]). Major PRCs are histone-modifying complexes that confer different and even counteracting enzymatic activities, which mediate gene repression. In animals and plants, PRC1 is an E3 ubiquitin ligase complex that catalyzes histone 2A lysine ubiquitination (H2AKub: K118 in Drosophila, K119 in vertebrates, and K121 in plants) [6,7,8], and PRC2 is a histone methyltransferase (HMT) complex that catalyzes histone 3 lysine 27 methylation (H3K27me) [9]. Some PRCs catalyze H2Aub removal. In animals, the PR-DUB (polycomb repressive deubiquitinase) complex catalyzes histone H2A deubiquitination, which contributes to gene repression (reviewed in the work of [10,11]). Similarly, in plants, H2A deubiquitinases UBP12 and UBP13 are implicated in polycomb repression [12,13], but the composition of associated protein complexes remains enigmatic. Additional PRCs have been described in animals, such as Drosophila pleiohomeotic repressive complex (PhoRC) that does not confer enzymatic activity ([14], reviewed in the work of [15]). As histone modifications introduced by PRCs are heritable during mitotic cell divisions (reviewed in the work of [16,17,18]), PRCs provide an epigenetic memory system required for stable cell identity, for an adequate response to external cues, and even for stable repression of genomic repeats (reviewed in the work of [19]). In line with its function in cell identity maintenance in animals, PRC2 dysfunction is frequently associated with cancer development and PRC2 is a potent target for anticancer therapy (reviewed in the work of [20,21,22]).

**Figure 1:**
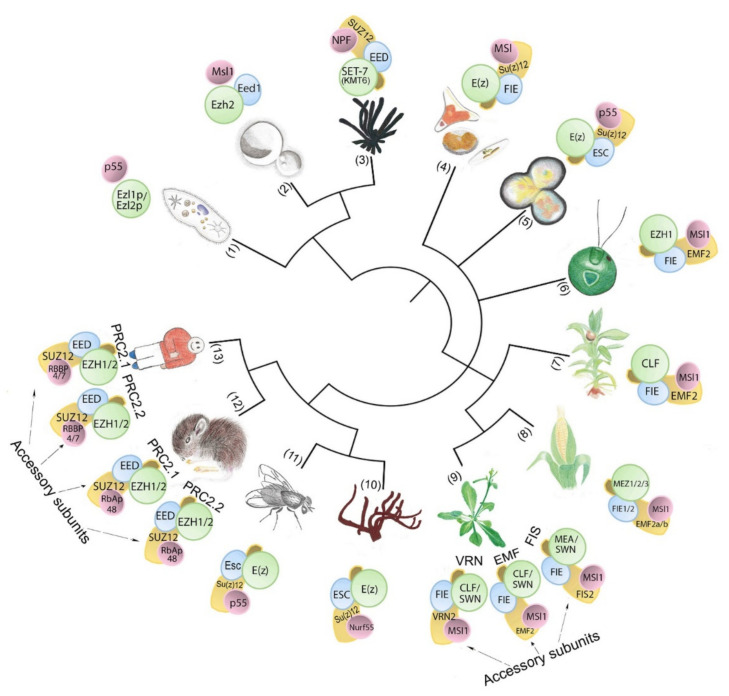
PRC2-core subunit diversity in unicellular and multicellular organisms. The number of subunit homologs and putative PRC2 complexity hypothetically increases with body plan complexity. Numbers in brackets indicate species (**1**) *Paramecium tetraurelia;* (**2**) *Cryptococcus neoformans;* (**3**) *Neurospora crassa;* (**4**) *Phaeodactylum tricornutum;* (**5**) *Cyanidioschyzon merolae;* (**6**) *Chlamydomonas reinhardtii;* (**7**) *Physcomitrium patens;* (**8**) *Zea mays;* (**9**) *Arabidopsis thaliana;* (**10**) *Amphimedon queenslandica;* (**11**) *Drosophila melanogaster;* (**12**) *Mus musculus;* (**13**) *Homo sapiens.*.

PRC1 and PRC2 are conserved in animal and plant models. PRC1 was long considered to be animal specific since a homolog of Polycomb (Pc), the Drosophila PRC1-defining protein subunit [23], is missing in plants. Later, orthologs of PRC1 catalytic subunits RING1 (Really Interesting New Gene 1) and BMI1 (B cell-specific Moloney murine leukemia virus integration site 1) were identified in plants and worms [24]. RING1 and BMI1 were shown to be indispensable for plant development and required for H2Aub [7,25,26]. RING1 and BMI1 orthologs, as well as plant-specific PRC1 subunits, have been found in different plants species ([27], reviewed in the work of [28]). Hence, PRC1 is present in plants, although its core composition differs from animals and may have originated through convergent evolution (reviewed in the work of [28,29,30]). In contrast to PRC1, PRC2 core composition and H3K27me activity are well conserved throughout eukaryotic lineages, and PRC2 is hypothesized to have emerged through divergent evolution [31]. The evolution of PRC2 has been shaped by genome duplication and subfunctionalization, and the number of different PRC2 complexes tends to rise with increasing body plan complexity (Figure 1) ([32], reviewed in the work of [15,33,34]). In animals, PRC2 catalyzes H3K27 mono, di, and trimethylation (i.e., H3K27me1, H3K27me2, H3K27me3), but in flowering plants, it catalyzes H3K27me3 ([35,36], reviewed in the work of [9,18]). In model species of both animals and plants, H3K27me3 is largely associated with transcriptional silencing of developmental genes ([37,38], reviewed in the work of [39,40]). PRC2 composition, its biochemical and developmental functions are well studied in animal and in flowering plant model species, and we refer to recent reviews for detailed information [41,42,43,44,45,46,47,48,49]. Here, we bring a comparative overview of PRC2 core composition and biochemical function in different eukaryotic groups, with focus on the green lineage, to highlight emerging concepts of PRC2 evolution.

## 2. Features of PRC2 Core Composition and Function Are Conserved in Animal and Plant Models

**Figure 2:**
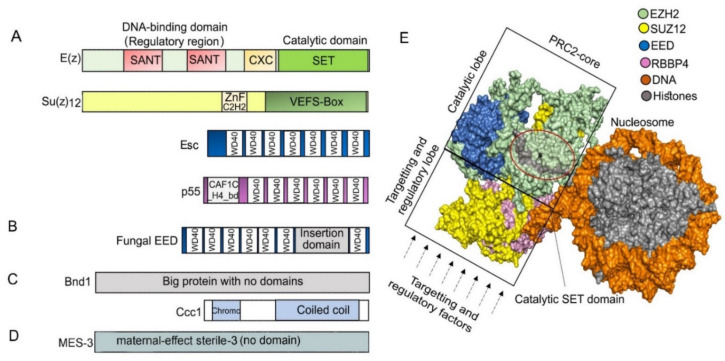
(**A**) Typical domain architecture of PRC2 core subunits in *D. melanogaster*. The catalytic subunit E(z) contains SANT (SWI3-ADA2-N-CoR-TFIIIB), CXC (pre-SET domain with C-X(6)-C-X(3)-C-X-C motif), and SET (Su(var)3–9, enhancer of zeste, and trithorax domain) domains, and it catalyzes H3K27me through the SET domain. Su(z)12 contains ZnF_C2H2 (cysteine 2-histidine 2 zinc finger domain) and VEFS (VRN2-EMF2-FIS2-Su(z)12) domain and is responsible for assembling the PRC2 complex, while Esc (WD40 repeat protein) helps to stabilize and enhance E(z) activity. P55 (WD40 repeat protein) can bind to histones and Su(z)12 and is necessary for nucleosome interaction. (**B**) Most of the studied fungal Esc homologs EED carry a long insertion domain in their C-terminal part. (**C**) While Su(z)12 is missing, subunits Bnd1 (Big protein with no domains) and Ccc1 (Chromodomain and a coiled coil region) are present in *Cryptococcus neoformans.* (**D**) Su(z)12 is missing in *C. elegans,* but the protein MES-3 (no detectable domains) is a part of the core PRC2. (**E**) PRC2 core structure. Surface depiction of human PRC2 core complex was created using PyMOL 2.4.1 [85] based on PDB ID: 6WKR [86]; only core subunits are shown. In a catalytically active complex, the EED subunit is encircled by EZH2, and the C-terminal VEFS domain of SUZ12 is sandwiched between the EED and EZH2-SET domain [87]. The region in the circle highlights the lysine binding channel (gray) through which a methyl group is transferred from the cofactor S-adenosylmethionine (SAM)) to the substrate H3 peptides. The catalytic or targeting and regulatory functions of mammalian PRC2 are separated into two regions within the structure: into the top catalytic lobe (containing EZH, VEFS domain of SUZ12 and EED) and the bottom targeting and regulatory lobe (containing RBBP4/7 and N-terminal SUZ12) (reviewed in the works of [42,46,84,88]).

The core of PRC2 is generally composed of four protein subunits that are conserved in multicellular eukaryotic model organisms (Figure 1, Table 1, Supplementary Table S1). The four subunits enhancer of zeste (E(z)), suppressor of zeste 12 (Su(z)12), extra sex combs (Esc), and nucleosome remodeling factor (Nurf55, also called p55) are the essential PRC2 components present in *D. melanogaster* (Figure 2) ([50], reviewed in the work of [15]). E(z) contains the catalytic SET domain (Suppressor of variegation 3–9 (Su(var)3–9), Enhancer of zeste (E(z)), Trithorax (Trx) domain), which is responsible for the HMT activity ([50], reviewed in the work of [51]). Su(z)12 is a VEFS (VRN2-EMF2-FIS2-Su(z)12) domain-containing protein, and Esc and p55/Nurf55 are two WD40 repeat (WDR) domain proteins. Su(z)12, Esc, and Nurf55 are non-catalytic subunits that are crucial for PRC2 catalytic activity ([52,53,54,55], reviewed in the work of [56]). PRC2 containing all four core components is 1000 times more active than the E(z) subunit alone [50,57]. Esc and Su(z)12 play critical roles in stimulating the HMTase activity of E(z) [58,59], and the loss of Esc impairs global H3K27me in Drosophila embryos [52,57]. Homologs of Drosophila PRC2 subunits are conserved in mammals, fungi, plants, red algae, and diatoms (Figure 1, Table 1, Supplementary Table S1) ([60,61,62,63,64,65], reviewed in the work of [66,67,68,69]). In mammals, the homologs of E(z), Su(z)12, Esc, and p55 are EZH1 and EZH2, SUZ12, EMBRYONIC ECTODERM DEVELOPMENT (EED), and RETINOBLASTOMA BINDING PROTEINS (RBBP4 or RBBP7), respectively. Minimal PRC2 in mammals comprises the EZH1/2, EED, and SUZ12 subunits in a 1:1:1 stoichiometry [70], and the trimeric core is responsible for H3K27me1/2/3 (reviewed in the work of [71]). RBBP4 or RBBP7, which display 92% sequence identity, are frequently found together in the same complex, but they may differ functionally, as only RBBP4 is involved in maintaining stem cell identify ([72], reviewed in the work of [73]). Core PRC2 possesses lower activity and/or affinity to target sites unless associated with accessory proteins (reviewed in the work of [56]) that define subtypes of PRC2s ([74], reviewed in the work of [75,76]). In humans, two PRC2 subtypes are present based on association with accessory proteins, namely PRC2.1 (contains polycomb-like 1–3 (PCL1/2/3), elongin BC and PRC2 (EPOP), PRC2-associated LCOR isoform 1/2 (PALI1/2)), and PRC2.2 (contains jumonji and AT-rich interaction domain-containing 2 (JARID2), adipocyte enhancer-binding protein (AEBP2)) (reviewed in the work of [10]). The molecular structures have been determined for Drosophila, human, mouse, and the fungus *Chaetomium thermophilum* PRC2 (Figure 2) ([60,77,78,79,80], reviewed in the work of [34,81,82,83,84]).

The flowering dicot model plant *Arabidopsis thaliana* has three E(z) paralogs (CURLY LEAF (CLF), SWINGER (SWN) and MEDEA (MEA)), three Su(z)12 paralogs (EMBRYONIC FLOWER 2 (EMF2), VERNALIZATION 2 (VRN2), and FERTILIZATION INDEPENDENT SEED 2 (FIS2)), one ESC homolog (FERTILIZATION INDEPENDENT ENDOSPERM (FIE)), and five p55 homologs (MULTICOPY SUPPRESSOR OF IRA (MSI1–MSI5)), of which only MSI1 is known to be present in PRC2 ([102,130,131], reviewed in the works of [29,132]). SWN and CLF play significant roles during vegetative development and phase transitions ([133,134,135,136,137,138], reviewed in the work of [47,48]), while MEA is required during gametophyte development and early embryogenesis [131,139]. Three PRC2 complexes have been identified in Arabidopsis defined by the presence of respective Su(z)12 homologs: EMBRYONIC FLOWER (EMF), VERNALIZATION (VRN), and FERTILIZATION INDEPENDENT SEED (FIS). The EMF and VRN complexes are associated with either CLF or SWN, and the FIS complex contains MEA or SWN as a catalytic subunit ([140,141,142], reviewed in the work of [3,69]). In *A. thaliana*, accessory subunits that can physically interact with core PRC2 subunits have been identified, including several transcription factors, such as the ASYMETRIC LEAVES (AS), TELOMERE REPEAT BINDING FACTORS (TRBs), VIVIPAROUS1/ABI3-LIKE (VAL), additional PRC-chromatin-associated enzymatic or scaffold proteins, such as LIKE HETEROCHROMATIN PROTEIN 1 (LHP1), VERNALIZATION INSENSITIVE 3 (VIN3), VRN5, PWWP-DOMAIN INTERACTOR OF POLYCOMBS 1 (PWO1), INCURVATA11 (ICU11), components of the E3 ubiquitin ligase complexes, components of the DNA replication and chromosome segregation machineries (CTF4) or domesticated transposases (ALP1, ALP2) ([143,144,145,146,147,148,149], reviewed in the work of [3,33,45]). In spite of the well-described impact of PRC2 function on plant development, plant PRC2 composition and potential subcomplex function remain enigmatic. Additionally, structural and detailed biochemical information on plant PRC2 is not yet available, limiting detailed insight into PRC2 subunit interaction and mechanism of action in plants.

**Figure 3:**
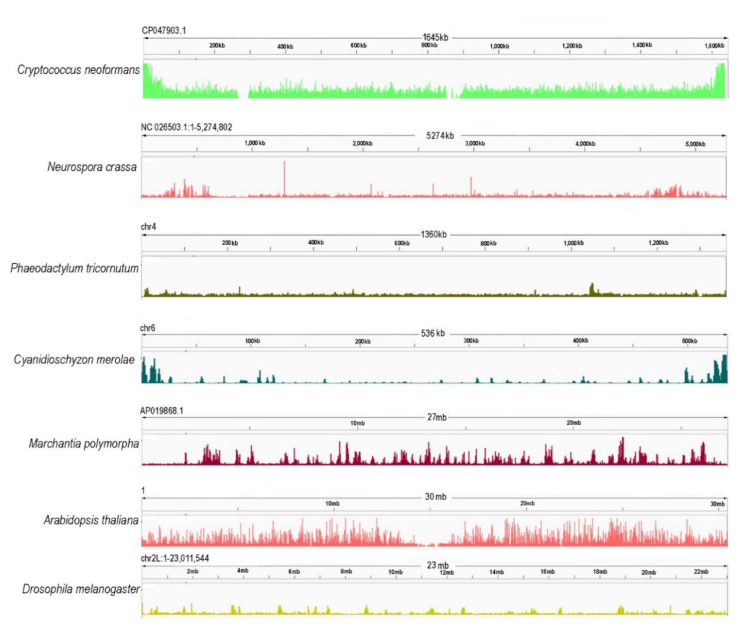
Chromosome-wide distribution of H3K27me3 in different model species [*Cryptococcus neoformans* (ASM1180120v1/PRJNA261445) [64], *Neurospora crassa* (NC12/PRJNA192863) [111], *Phaedactylum tricornutum* (ASM15095v2/PRJNA282957) [165], *Cyanidioschyzon merolae* (ASM9120v1/PRJNA362822) [63], *Marchantia polymorpha* (ASM993635v2/PRJNA553138) [166], *Arabidopsis thaliana* (TAIR10/PRJNA277409) [167], *Drosophila melanogaster* (PRJNA379297) [168]. Genome reference and accessions are given in brackets. Here, the publically available data were downloaded, cleaned by removing the library adapters, small reads (30), and low-quality (20) reads using Trim galore [169]. To map the sequenced data, Bowtie2 [170] was used with default parameters. Only mapped reads were kept with a quality threshold of 25. The filtering was performed using SAMTOOLS [171]. For the visualization of the data, IGV [172] was used, and for the parsing of the BAM files to BW files, BamCoverage—deeptools [173].

In animals, PRC2 catalyzes H3K27me1/2/3 [50], while in plants, H3K27me1 in centromeres and pericentromeres is catalyzed by the ARABIDOPSIS TRITHORAX-RELATED HMTs (ATXR5 and ATXR6) [150]. H3K27me3 is, therefore, the conserved hallmark of PRC2 enzymatic activity (Figure 3, Supplementary Table S2). H3K27me3 in *D. melanogaster* occupies broad domains that typically span more than 10 kb [151]. In mammals, H3K27me3 has two distribution patterns: large domains (>100 kb) encompassing the Hox loci and smaller domains of a few kilobases (reviewed by the authors of [75]). In addition to genic loci, H3K27me3 and PRC2 reside in poised enhancers (PEs) that often associate with bivalent genes in vertebrate pluripotent cells [152]. While PRC1 contributes to the PE marking globally [152] and also targets active enhancers in cancer cells [153], PRC2 is involved at PEs at specific loci [152]. Unlike in animals, the distribution of H3K27me3 in *A. thaliana* is usually limited to single genes, with the modification covering proximal parts of promoters and gene bodies with average enrichment regions of 1–1.5 kb [37,38,154,155]. Gene-limited distribution is also found in metabolic gene clusters that are organized in an operon-like manner [156]. In *A. thaliana*, H3K27me3 decorates approximately 20–30% of all protein-coding and miRNA genes [37,38,157] that are associated with plant development, hormone metabolism and response, but also with nutrient homeostasis [158,159], stress response ([160,161], reviewed in the works of [5,162,163]) or primary and secondary metabolism [156,161]. The potential for targeting is nevertheless more extensive, as 64% of protein-coding genes were identified as H3K27me3 targets when combining different timepoints of *A. thaliana* shoot apical meristem development during the transition to flowering [164].

PRC2 itself lacks sequence-specific DNA-binding ability and therefore relies on accessory proteins for targeting specific loci. Several mechanisms by which PRC2 is recruited to the chromatin targets have been identified. Among these are transcription factor site-specific recruitment, interaction with RNAs, or association with chromatin features (reviewed in the work of [88]). In *D. melanogaster*, PRC2 is recruited to DNA targets by different transcription factors that interact with sequence motifs known as polycomb response elements (PREs) ([174,175], reviewed in the work of [176]). PREs in Drosophila are found in proximal promoter regions of developmental genes. They typically span about 1.5 kb [177] and contain numerous binding sites for a variety of DNA sequence-specific binding factors, such as the GTGT-motif-binding Combgap (Cg) [178], which together mediate PRC2 recruitment to target sites (reviewed in the work of [75]). Typical fly-like PREs are not found in mammals, and even though several mammalian PREs have been reported [179], they are not evolutionarily conserved. CpG islands, hypomethylated CG-rich chromatin regions of 1–2 kb, are associated with PRC2 recruitment in mammals, where accessory subunits rather than transcription factors mediate the recruitment ([180,181,182], reviewed in the work of [71]). Transcription factor-bound PRE-like elements also contribute to PRC2 recruitment in *A. thaliana* (reviewed in the work of [5]). Following the identification of PRC2-recruiting cis elements in the promoters of the KNOX genes [183] and the LEC2 gene [184], PRE-like elements (including GAGA motif, W-box, RY motif, GCCGCC motif, telobox motif, and others) bound by transcription factors were shown to contribute to PRC2 recruitment genome-wide [185]. Several transcription factors have been demonstrated to interact directly with PRC2 subunits to mediate the complex’s recruitment [144,146,183,186]. For instance, transcriptional repressors VIVIPAROUS1/ABI3-LIKE1 (VAL1) and VAL2 or TELOMERE REPEAT-BINDING FACTORS (TRBs) physically interact with SWN and CLF to recruit PRC2 to specific sequence motifs present in the promoters of target genes [144,146,187]. PRC2 recruitment through interaction with trans-acting factors that recognize cis elements is thus conserved in both plant as well as animal models (reviewed in the work of [45]).

Noncoding RNAs (ncRNAs), in particular long noncoding RNAs (lncRNAs), and nascent RNAs have also been implemented in PRC2 binding and recruitment to target sites (reviewed in the work of [188,189]). The formation of DNA-RNA hybrid structures (R-loops) is suggested to promote PRC2 recruitment in mammalian cells [190]. R-loops have been found at a variety of polycomb target gene loci, mostly found adjacent to the promoters (reviewed in the work of [191]), and interestingly, R-loop formation was detected at about one-third of PREs in Drosophila embryos [192], suggesting conservation of the mechanism. R-loops have been shown to positively and negatively impact recruitment, and their role is discussed [190,193]. lncRNAs are also known to contribute to PRC2 recruitment in *A. thaliana*. For instance, *COLD ASSISTED INTRONIC NONCODING RNA* (*COLDAIR*), *COOLAIR*, and *COLDWRAP* act in repressing FLOWERING LOCUS C (FLC) transcription by H3K27me3 [194,195] and the lncRNA *APOLO* contributes to LHP1 recruitment, H3K27me3 enrichment, and chromatin looping at the *PINOID* (*PID*) locus that encodes a key polar auxin transport regulator [196].

**Figure 4:**
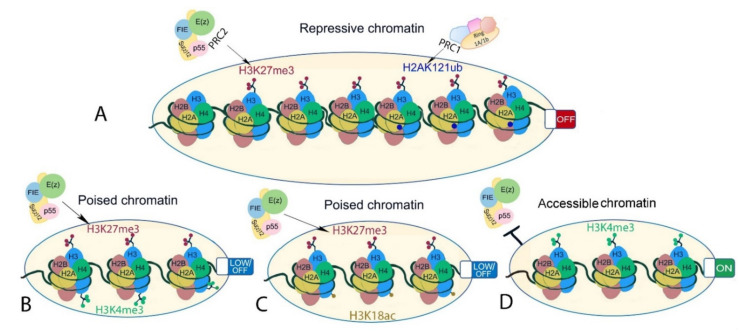
Different chromatin states associated or interacting with H3K27me3 in plants. (**A**) Facultative heterochromatin-inaccessible H3K27me3-marked chromatin (left) and inaccessible but permissive H3K27me3- and H2AK121ub-marked chromatin (right). (**B**,**C**) bivalent chromatin is marked by histone modifications with opposing effects on gene transcription. Examples are (**B**) H3K27me3-H3K4me3 and (**C**) H3K27me3 and H3K18ac. These are poised states where gene transcription is readily initiated upon stimuli. (**D**) Euchromatin showing chromatin state at transcriptionally active genes that is known to interfere with PRC2-mediated H3K27me3 deposition on the same histone tail in *A. thaliana*.

Last but not least, PRC2 recruitment and activity are prevented or promoted by its interaction with the existing chromatin environment (Figure 4). Several pre-existing chromatin modifications, including H3K27ac, H3K4me3, H3K36me2/3, and cytosine methylation in CpG islands, prevent PRC2 recruitment and/or inhibit its activity ([77,180,197,198,199,200], reviewed in the work of [201]). In addition to opposing PRC2, H3K36me3 may promote H3K27me3. In mouse embryonic stem cells, Phf19, a PCL ortholog, binds to H3K36me2/H3K36me3, recruiting PRC2 and lysine demethylases to promote PRC2 activity [202]. Although biochemical evidence is limited in plants [198], H3K27me3 mainly occupies regions that are depleted for active chromatin modifications [154,155]. An exception to this is regions of bivalent chromatin where activating and repressive modifications co-localize to potentiate rapid change of gene transcription. In both plant and animals, the best well-described bivalent chromatin is marked by H3K27me3 and H3K4me3 ([155], reviewed in the work of [203]), but other active modifications may co-localize with H3K27me3 including H3K4me1 in *Brassica napus* [204], or H3K18ac in the camalexin biosynthesis genes in *A. thaliana* [205]. In mammals, H3K27me3 is promoted in a self-reinforcement loop. Pre-existing H3K27me3 is bound by EED, which allosterically stimulates PRC2 to methylate adjacent unmodified H3K27 and promote the spreading of H3K27me3 [87,206,207]. H3K27me3 binding activity has not been shown for the *A. thaliana* EED homolog FIE. Nevertheless, *A. thaliana* MSI1 interacts with LHP1, which binds H3K27me3, and through this may enable H3K27me3 spreading and/or post-replicative maintenance of H3K27me3 [102,208]. In addition, H2AKub, the catalytic product of PRC1, can act as a recruitment platform for PRC2 ([209,210], reviewed in the works of [45,211]). The human PRC2 cofactors JARID2 and AEBP2 bind to H2AK119ub, triggering a positive feedback loop [77] that ensures the maintenance of transcriptional repression. In animals as well as in plants, genome-wide deposition of H3K27me3 and H2Aub seem to be partially dependent on each other. In animal models (Drosophila, mouse, and human cells), most studies suggest that H2Aub modification is independent of H3K27me3 deposition, while H3K27me3 levels are decreased upon disruption of H2Aub (reviewed in the work of [212]). In *A. thaliana*, H3K27me3 overlaps with H2AK121ub at a subset of loci [26]. H2Aub is more widespread than H3K27me3, and its deposition is largely independent of PRC2 activity. In contrast, H3K27me3 depends on H2Aub at sites that carry both the marks, together indicating that PRC1 may be instructive for H3K27me3 in plants rather than vice versa [26]. Recently, three different chromatin states occupied either by H3K27me3 only, H2Aub only, or both modifications were described in *A. thaliana*, showing that accessibility increases from the inaccessible H3K27me3-only-marked chromatin to H3K27me3/H2Aub and H2Aub-only chromatin, that are mainly located at transcriptional hotspots [213]. This is in line with findings that H2Aub associates with responsive genes and its repressive function relies on H3K27me3 deposition and that H2A deubiquitination by UBP12 and UBP13 are required for stable H3K27me3-mediated repression [12,13]. Thus, while PRC1 and PRC2 activities both contribute to decreased chromatin accessibility, H3K27me3 seems to be a major contributor to stable gene repression within inaccessible chromatin regions in *A. thaliana*.

## 3. PRC2 Is Conserved throughout Evolution in Unicellular and Multicellular Eukaryotes

Despite their conservation in animal and plant multicellular model organisms, the absence of PRC2 in unicellular model organisms such as *S. cerevisiae* and *S. pombe* (reviewed in the work of [15,214]) and the requirement for the specification of cell identity led to the initial hypothesis that it was generally absent in unicellular models and evolved with multicellularity (reviewed in the work of [215,216]). Later, PRC2 subunit homologs were identified in the genomes of unicellular species of different eukaryotic supergroups, suggesting that PRC2 originated early in eukaryotic evolution [31,217] and could have been present already in the phylogenetically deduced putative reference biological state known as the “Last Eukaryotic Common Ancestor (LECA)” [218,219]. The absence of PRC2 in the model yeasts is currently attributed to secondary loss [31,75]. PRC2 and H3K27me3 distribution were described in the diatom *Phaeodactylum tricornutum* [165], the budding yeast *Cryptococcus neoformans* [64], and the red alga *Cyanidioschyzon merolae* [63], confirming PRC2 composition and functional conservation in unicellular representatives of diverse eukaryotic supergroups. The widespread presence of PRC2 subunits in unicellular species was demonstrated in an array of marine microalgae of diverse supergroups [65,103,217], further supporting the conservation and early origin of PRC2. PRC2 evolution is thought to have been shaped by genome duplication and subunit diversification, as the diversity of PRC2s tends to rise with increasing body plan complexity (Figure 1) [89,220,221]. Below, we bring a brief overview of known aspects of PRC2 function in diverse clades of eukaryotic supergroups including SAR (Stramenopila, Alveolata, Rhizaria), ciliates (Alveolata) and diatoms (Stramenopila), Ophistokonta, fungi and simple multicellular animals, red algae (Rhodophyta) and the green lineage (Viridiplantae).

### 3.1. PRC2 in Stramenopiles, Alveolates and Rhizaria (SAR)

PRC2 subunits and H3K27me3 are present in species of the SAR groups, although secondary absence may be common in the diverse taxa [217]. PRC2 subunits and H3K27me3 are found in Rhizaria [65,103], but no further information on PRC2 function is available in this group. Dinoflagellate (Alveolata) genome that is organized in a liquid crystalline structure [222] encodes divergent H3 variants and multiple SET domain proteins [223]. PRC2, however, seems to be absent here with the exception of the Esc homolog in *Symbiodinium* and *Alexandrium* species [103], perhaps reflecting the absence of structural nucleosomes and limited involvement of Dinoflagellate histones in heterochromatinization [65,223,224]. In the SAR groups, PRC2 has been best well studied in ciliates (Alveolata) and the model diatom *Phaeodactylum tricornutum* (Stramenopila).

Ciliates (Alveolata) are widely used in evolutionary studies because of the early evolutionary divergence [225]. Cells of ciliates contain two nuclei: the diploid germline micronucleus (MIC), which remains transcriptionally silent during vegetative growth and provides genetic material transferred to sexual progeny, and the somatic polyploid macronucleus (MAC), which provides for transcription during vegetative growth. The macronucleus genome undergoes complex rearrangements, which include small RNA (“scan” RNA—scnRNA)-guided heterochromatinization and elimination of repetitive sequences (reviewed in the work of [226]). The heterochromatinization event involves deposition of H3K9me2/3 and H3K27me3, RNAi, and the activity of the E(z) homolog EZL1 [227,228,229,230]. EZL1 in *Tetrahymena thermophila* is a component of the EZL1 complex that comprises subunits homologous to animal PRC1 as well as PRC2 subunits [107]. EZL1 complex interacts with ENHANCED MIRNA ACTIVITY 1 (EMA1), a helicase implicated in RNA interference, via scnRNA-mediated anchoring to nascent ncRNA transcripts, which is thought to be critical in EZL1 recruitment to chromatin [107]. Importantly, EZL1 in *T. thermophila* and *Paramecium teraurelia* carries out methylation of H3K27 as well as H3K9, indicating dual substrate specificity, which is likely to be conserved in ciliates [106,107]. In both ciliate models, EZL1 is required for the repression of TEs, marked by H3K9 and H3K27 methylation [106,107]. Although H3K9me3 and H3K27me3 marks are catalyzed by distinct HMTs in animal and plant models, studies in ciliates indicate that these epigenetic marks may share a common evolutionary history. Interestingly, in addition to H3K27, H3K9 was also described as a substrate for PRC2 in initial biochemical studies in Drosophila and mammals [50,231,232,233]. Even though this remains unresolved, it raises the possibility that under specific conditions, H3K9 may serve as a substrate in distant E(z) homologs [217].

Diatoms are photosynthetic secondary endosymbionts (microalgae) found throughout marine and freshwater environments. The pennate diatom *Phaeodactylum tricornutum* (Stramenopila) is a well-studied model species. PRC2 and H3K27me3 are conserved in *P. tricornutum* [65,165]. H3K27me3-marked regions cover about 14% of the *P. tricornutum* genome, with H3K27me3 being particularly abundant at TEs [165]. *P. tricornutum* has a variety of morphologies (fusiform (FM) most stable morphotype, triradiate (TM), oval (OM), and cruciform (CM)) that are determined by PRC2 and H3K27me3 [65]. Therefore, PRC2 and its associated mark H3K27me3 are proposed to modulate cell differentiation and cell identity also in unicellular organisms [65]. This is the first evidence of H3K27me3 influencing cell morphology in a unicellular eukaryote, and this function may have been retained and/or become dominant in multicellular plants and animals. It is interesting to note, however, that in contrast to PRC2 conservation and requirement for cell identity specification in the unicellular diatom, multicellular brown algal species of the Stramenopile group (*Ectocarpus siliculosus* Ec32, *Cladosiphon okamuranus*, *Nemacystus decipiens*, and *Saccharina japonica*) lack H3K27me3 and PRC2 core subunits except p55, as well as PRC1 components [104], indicating that alternative mechanisms operate in cell identity specification in these species.

### 3.2. PRC2 in Ophithokonts

PRC2 is well studied in fungi (reviewed in the works of [109,214,234]), and reports are emerging that describe PRC2 function in simple multicellular animals [114,115]. Fungi are marked by secondary loss of PRC2 in multiple species. In fungal clades such as Schizosaccharomycetales, Saccharomycotina, and Eurotiomycetidae, PRC2 subunits seem to be absent, and H3K27 methylation is not detected in *S. cerevisiae*, *S. pombe,* and *Candida albicans* (reviewed in the work of [214]). H3K27me3 was first identified in the filamentous ascomycete fungus *Neurospora crassa* [235], and homologs of animal PRC2 subunits and H3K27me marks were identified in other ascomycete and basidiomycete species (reviewed in the work of [68,214]). Crystal structure of PRC2 is available for *Chaetomium thermophilum*, thermophilic pathogenic ascomycete [79]. CtPRC2 has structural similarities to its human equivalent, implying that fungal and animal PRC2s are structurally comparable [236]. However, substantial differences exist between metazoan and fungal PRC2 core subunits at the protein sequence level. Fungal E(z) homologs (KMT6 proteins) are longer and display limited sequence conservation outside the CXC and SET domains compared to metazoan E(z) homologs, but the sequence is conserved among fungal KMT6 proteins, supporting conservation of function and protein-protein interactions in fungi [110]. Esc/EED homologs in studied fungi are distinguished by a long “insertion domain” of unknown function near the C-terminal end, that is absent in plants and animals (reviewed in the work of [234]). SUZ12 homologs can be missing in some fungal species (e.g., *C. neoformans*) but can be functionally substituted by other proteins (reviewed in the work of [109,214,234]).

PRC2 is well studied in the filamentous ascomycete *Neurospora crassa* and the basidiomycete *Cryptococcus neoformans,* and H3K27me3 distribution has been studied in the ascomycetes *Fusarium graminearum*, *F.* *fujikuroi,* and *Podospora anserina* [64,110,112,237,238]. In the filamentous ascomycete *Neurospora crassa*, all core PRC2 subunits are found, including E(z) homolog SET-7 (KMT6), EED, SU(Z)12, and the p55 homolog NPF (*Neurospora* protein 55), which are all required for H3K27me3 [111]. In addition, a previously unknown PRC2 Accessory Subunit (PAS) was recently discovered, *N. crassa* [112]. NPF and PAS are required for H3K27 methylation in subtelomeres, but not at internal PRC2 target sites [111,112]. PAS has homologs in other fungal species, indicating that it is not unique to *N. crassa*. In a number of fungal lineages (e.g., Sordariomycetes and Leotiomycetes), PAS homologs are predicted to alter the distribution of H3K27 methylation and underlying gene repression. H3K27me3 covers around 7% of the *N. crassa* genome and spans large domains, which are predominantly located in the proximity of telomeres and include hundreds of transcriptionally inactive genes [111]. H3K27me3 in telomeres resides in gene-containing regions, and it is neighbored by non-overlapping H3K9me3 marking gene depleted, DNA-methylated regions [111,235]. Interestingly, H3K27me3-marked loci represent only a small subset of inaccessible regions of chromatin as determined by ATAC-seq, and H3K36me, rather than H3K27me3, is a predictive mark of chromatin inaccessibility in gene-rich regions in *N. crassa* [239].

Different extent of H3K27me3 marking is observed in other ascomycete species that have been studied [110,237,238]. *Fusarium graminearum* is an ascomycete pathogen that causes fusarium head blight in wheat and barley. H3K27me3 locates in broad domains mainly to subtelomeric regions, and it is deposited by the E(z) homolog KMT6. In contrast to *N. crassa*, about 30% of the genome is marked by H3K27me3. About 75% of silent genes are enriched in H3K27me3, and among these are major secondary metabolic gene clusters [110]. A similar distribution of H3K27me3 is observed in the rice pathogen *Fusarium fujikuroi*, where secondary metabolite clusters frequently located in subtelomeric regions are primarily targeted [237]. In the filamentous ascomycete *Podospora anserina*, H3K27me3 covers approximately 20% of the genome and, similarly to other ascomycetes, it is located in subtelomeric regions [238]. It is interesting to note that while deletion of KMT6, EED, or SUZ(12) in *N. crassa* has no visible phenotype effect [111], KMT6 dysfunction in *Fusarium* species and *P. anserina* results in activation of secondary metabolic genes and severe growth and developmental defects [110,237,238]. In *P. anserina*, a tight link between constitutive and facultative heterochromatin is observed. H3K27me3 is exclusive with H3K9me3 in gene-rich regions, but the two marks overlap and are interdependent in repeats [238].

Similar H3K27me3 distribution as in *N. crassa* is found in the basidiomycete fungus *C. neoformans*. Here, H3K27me3 occupies approximately 5% of the genome and is concentrated in subtelomeres, creating broad gene-repressing domains [64]. *C. neoformans* PRC2 comprises a five-subunit functional core composed of an E(z) homolog, two WD40 domain proteins (Esc/EED ortholog Eed1 and p55 ortholog Msl1), and two additional subunits: Bnd1 (Big protein with no functional domains) and a coiled coil chromodomain-containing subunit Ccc1 that recognizes H3K27me3 [64]. The Su(z)12 subunit is missing in *C*. *neoformans* [31,64,240], and, instead of the Su(z)12-containing lobe, the Ccc1 and Bnd1 may facilitate targeting [64]. Indeed, the absence of the Ccc1 subunit causes the redistribution of H3K27me3 into H3K9-methylated centromeric regions. The redistribution depends on the H3K9me2 HMT Clr4 and was proposed to be attributed to the latent affinity of Eed1 to HK9me2 that is potentiated upon Ccc1 depletion [64]. Ccc1 and Bnd1 are more restricted in their conservation, suggesting the presence of specific PRC2 complexes in some fungal species (reviewed in the work of [214,234]).

Despite the general presence of PRC2 in metazoans, *Capsaspora owczarzaki*, the closest known unicellular metazoan relative, lacks H3K27me3 marks and PRC2 complex proteins [113]. PRC2 subunit homologs and H3K27me3 are found in sponges (phylum Porifera), ancient animal species that split from other multicellular eukaryotes (metazoans) ~600 million years ago, earliest among surviving metazoan lineages ([241], reviewed in the work of [242]). PRC2 subunit homologs and H3K27me3 are conserved in the marine sponge *Amphimedon queenslandica* [114]. Like in Drosophila, *A. queenslandica* PRC2 complexes are likely to be recruited via PRE-like sequences that contain conserved binding motifs similar to GAGA and Kruppel-like motifs, as well as transcription factors similar to homeodomain-containing developmental regulators (e.g., Irx-family members), suggesting conservation of targeting and recruitment [114]. PRC2 subunits are also conserved in *Hydra vulgaris*, a freshwater polyp and regeneration model [115]. In *H. vulgaris*, the transcription factor Yin Yang 1 (YY1), a ubiquitous mammalian transcription factor that plays a crucial role in the development of the central nervous system (reviewed in the work of [243]), has an evolutionary conserved role in PRC2 recruitment for targeted gene regulation [116].

In the model nematode *Caenorhabditis elegans*, repressive H3K27me3 was detected on about 70% of embryonic histones, with a higher proportion of the modification located in chromosome arms [244,245]. PRC2 is conserved, with E(z) homolog MES-2 and ESC homolog MES-6. Su(z)12 homolog is missing but a *C. elegans*-specific core component MES-3 is present [122,123,246,247]. Like the Bnd1 subunit in *C. neoformans* PRC2 [64], the MES-3 component lacks any specific domain or motif and appears to be unrelated to any other polycomb proteins [57,248]. MES-3 might have emerged in *C. elegans* PRC2 to substitute the function of Su(z)12, despite the absence of sequence similarity [57]. Su(z)12 may therefore represent a dispensable component of PRC2 in some species.

### 3.3. PRC2 in Cryptophytes, Red Algae, and the Green Lineage

PRC2 subunit homologs have been identified in cryptophytes [103] and in a number of unicellular red algae [31], but information on H3K27me3 distribution or PRC2 function remains mostly unknown. PRC2 is nevertheless studied in *Cyanidioschyzon merolae* [63], a red alga with a small low-repetitive genome (16 Mb), which makes it a suitable model for studying chromatin repression in an evolutionary context [249]. In *C. merolae*, H3K27me3 is predominant at telomeres and the subtelomeric region of the chromosomes and has a particular preference for intein-containing genes responsible for protein splicing [63].

Homologs of the PRC2 subunits have been identified in the genomes of representative species of the green lineage, including chlorophyte algae and land plants, confirming the evolutionary conservation of the complex (Table 1, Supplementary Table S1) ([89], reviewed in the works of [69,250]). Still, our insight into PRC2 evolution in the green lineage is limited (reviewed in the work of [250]), and most of the current understanding of plant PRC2 composition or function comes from studies in the dicot flowering plant model *Arabidopsis thaliana* (reviewed in the work of [3,18,45,47,101,251,252,253]) and monocot crop species such as rice (*Oryza sativa*) [91,254], maize (*Zea mays)* [94,255], and, more recently, bread wheat (*Triticum aestivum*) [97]. Information on PRC2 structure and function in the green lineage outside of angiosperms is scarce; however, a recent study in Norway spruce (*Picea abies*) brought first insights into gymnosperm PRC2 function [92]. Conservation of PRC2 subunits in bryophytes [89] together with the determination of their developmental roles [61,62,256] and recent elucidation of H3K27me3 distribution [166,257] brought first insights into PRC2 function in non-vascular land plants. Studies of the chlorophyte alga *Chlamydomonas reinhardtii* [31] have highlighted the possible differences in PRC2 function in the green lineage.

The first PRC2 components, homologs of the E(z), were identified in *A. thaliana* in the late 1990s as developmental regulators and repressors of homeotic genes, revealing conservation of PRC2 between animals and plants [258,259]. PRC2-subunit genes have gone through several rounds of gene duplication and diversification. Gene duplication followed by neo-functionalization is most apparent in the evolution of PRC2 in the genomes of angiosperms [89,91,93,94,220,260]. E(z) homologs are found throughout the green lineage [31,89], clustering into four clades, one comprising chlorophyte algae E(z) homologs and three land plant (Embryophyta) clades defined by the *A. thaliana* E(z) paralogs CLF, SWN and MEA [89]. CLF orthologs are most ancient, representing the only E(z) homologs in Bryophytes (*Physcomitrium patens*), Lycopodiophytes (*Selaginella moellendorffii* primitive spikemoss), or gymnosperms [91,221,261,262]. SWN orthologs are only identified in angiosperms (both in monocots and dicots). SWN is already found in the basal angiosperm species *Amborella trichopoda*, suggesting that SWN could have emerged with the separation of angiosperms and gymnosperms [92,261]. MEA is a shorter paralog of SWN that originated by duplication of SWN during the α whole-genome duplication (αWGD) and neofunctionalization in Brassicaceae [89,91,220,221]. Likewise, Su(z)12 homologs are found throughout the green lineage [89,90] with putative loss in *S. moellendorffii* and *Volvox carteri* [31,89]. Su(z)12 homolog found in the chlorophyte alga *C. reinhardtii* forms a separate clade from homologs in land plants. In land plants, EMF2 homologs are the most ancient and are found in non-vascular plants as well as in seed plants [90]. VRN2 homologs have only been reported in flowering plants so far, and VRN2 is hypothesized to have evolved through the duplication of an ancestral EMF2-like gene [90,221]. FIS2 is found only in Brassicaceae, where it emerged with the αWGD by duplication of VRN2 [89,90,91,221]. VRN2 has an oxygen-sensitive degradation sequence (N-degron) at its N-terminus, and its degradation decreases (and abundance increases) in hypoxic and cold environments, implying that VRN2 N-degron plays a role in environmental adaptation [263]. The presence of proteolysis-initiating methionine-cysteine (MC) dipeptide in the N-terminus in all the studied VRN2 homologs across angiosperms, including monocots, and its absence in the Su(z)12 homologs of basal land plants or mammals, suggests that O2-sensitive Su(z)12 proteins are solely present in angiosperms [263]. In multiple angiosperms, including *Amborella trichopoda*, but not basal land plants (lycophytes and bryophytes), EMF2 contains internally located MC-dipeptide not subject to N-end rule degradation, supporting VRN2 origin by EMF2 duplication and N-terminal truncation [263]. The Esc homolog FIE is highly conserved throughout the green lineage, suggesting an ancient role within the PRC2 [31,89]. In contrast to dicot species that contain a single FIE copy, monocots including rice, maize, and sorghum have two Esc homologs [91]. The single chromosomal location of FIE genes in rice and sorghum indicates that the two homologs evolved via a tandem duplication event [91]. Several FIE subunit paralogs with distinct chromosomal positions have been identified in bread wheat, which could be attributed to the allohexaploid nature of the genome [97]. A direct interaction between Esc and E(z) homologs has been demonstrated in *A. thaliana* [220,264] and based on structural conservation, Esc homologs may interact with other PRC2-core subunits in a manner similar to that of Drosophila and mammals (reviewed in the work of [45]). Finally, the p55 homologs MSI1–5 are substantially conserved across the green lineage, forming three distinct clades in phylogram, separating MSI1, MSI2 and MSI3, and MSI4 and MSI5 [132]. Among them, PRC2-core subunit MSI1 is conserved in chlorophyte algae as well as in land plants [69,89].

In angiosperms, many genome-wide histone modification studies have been conducted using Brassicaceae species or crops (e.g., maize, rice, barley, and wheat) [38,265,266,267,268]. Similar to *A. thaliana*, about one-third of protein-coding genes were identified as H3K27me3 targets in *Brassica rapa*, including key flowering genes [269]. Similar genomic distribution of H3K27me3 is also seen in monocots, including maize (*Zea mays*), rice (*Oryza sativa*), or *Brachypodium distachyon* [265,267,270,271]. In gymnosperms, H3K27me3 distribution has been studied cytologically [272] and also genome-wide in *P. abies* [92]. H3K27me3 was identified to be consistently located in the mid-arm areas of almost all chromosomes in *Pinus sylvestris* and Norway spruce, which is comparable to other angiosperms [272]. In Norway spruce, H3K27me3 plays a crucial role in embryogenesis, suggesting a conserved role of PRC2 in cell fate determination [92]. H3K27me3 distribution in the moss *P. patens* [257] and in the liverwort *Marchantia polymorpha* [166] brought important insights into the targeting of PRC2 in bryophytes. In the protonema and gametophore of *P. patens*, H3K27me3 localizes around the transcription start sites (TSS), covering the promoter region and spreading into the gene bodies [257]. In this respect, the distribution of H3K27me3 in *P. patens* seems similar as in *A. thaliana*, which, however, contrasts with the situation in *M. polymorpha*. Here, H3K27me3 occupies 30% of constitutive heterochromatin, forming a chromatin state distinct from constitutive heterochromatin occupied jointly by H3K27me1, H3K9me1, and DNA methylation [166]. Unlike in other land plants, H3K27me3 in *M. polymorpha* is distributed in broad domains that overlay inactive genes and surrounding repeats or transposons. As identified by Hi-C, these genomic sites also represent trans-interacting regions within the B-compartment, and PRC2 was proposed to function in heterochromatin organization in *M. polymorpha* [166]. In addition to H3K27me3, H2Aub distribution was recently studied in *M. polymorpha.* H2Aub localizes mainly to gene bodies and promoter regions, where it is required for the recruitment of H3K27me3. At a subset of TE’s, H2Aub is connected to H3K27me3 enrichment, and its absence leads to H3K27me3 depletion [273], suggesting similar inter-dependence, such as in *A. thaliana*. In bryophytes, unlike in *A. thaliana* [155], TEs and constitutive heterochromatin modifications such as H3K9me1/2, H3K27me1 do not concentrate in centromeric and pericentromeric regions but are rather dispersed throughout the genome [166,274]. Despite this similarity in the repetitive genome organization, the distribution of H3K27me3 in *P. patens* and in *M. polymorpha* seems to differ. It will be of great interest to identify the differences between the PRC2 complexes in these species that now seem to stand at the breakpoint of PRC2 function evolution. The distribution of H3K27me in chlorophyte and streptophyte algae still remains enigmatic. An early study reported H3K27 mono-methylation (H3K27me) in the unicellular green alga *C. reinhardtii* [275]. Later mass spectrometry analyses in *C. reinhardtii* showed that histone H3 lysine 27 (H3K27) can be mono- or di-methylated, but H3K27 trimethylation was not confirmed and suggested to be either absent or present at significantly low levels [31]. Enrichment of H3K27me3 in *C. reinhardtii* appeared to be very low in a ChIP-seq study, where inter- and intragenic distribution of H3K27me3 was suggested [276]. Despite the uncertainties as to H3K27me3 presence, homologs of E(z), EMF2, FIE, and p55/MSI1 have been identified in silico in the *C. reinhardtii* genome [31,89]. Since homologs of ATXR5 or ATXR6 were not found in *C. reinhardtii*. CrE(z) may be hypothetically responsible for the mono- and di-methylation of H3K27 [31]. RNAi-mediated suppression of *C. reinhardtii* E(z) resulted in ectopic upregulation of repetitive transgenes and retrotransposons, providing the first evidence for PRC2 function in repressing genomic repeats in unicellular eukaryotes [31]. Notably, loss of H3K27me1 in *A. thaliana atxr5 atxr6* mutant also results in the reactivation of heterochromatic transposable elements [150]. Whether reactivation of repetitive elements in *C. reinhardtii* is primarily connected to the loss of H3K27me3 or H3K27me1/2 remains unclear.

Based on the currently available information, the predominating function of PRC2 in the green lineage seems to have shifted during evolution from the organization of constitutive heterochromatin (*C. reinhardtii* and *M. polymorpha*) to facultative repression of genes that respond to the internal and external cues (*P. patens*, angiosperms). In *P. patens* [61,62,256] as well as in flowering plants (reviewed in the work of [47,48,251,252]), PRC2 is required for correct timing and execution of developmental phase transitions. In particular, the alternation between sporophyte and gametophyte and repression of apogamy depends on PRC2 in *A. thaliana* [277,278,279] as well as *P. patens* [256], demonstrating an evolutionarily conserved role of the complex. In accordance, developmental genes in both *A. thaliana* (reviewed in the work of [47,48,251,252]) and *P. patens* [257] undergo H3K27me3 remodeling during developmental phase transitions. In *A. thaliana*, response to salt stress and nitrate or iron deficiency is modulated by PRC2 [158,159,160], implicating PRC2 in environmental response. In *P. patens*, drought stress was not associated with changes in H3K27me3 distribution [257], suggesting a limited impact of PRC2 in this response. Evolutionary conservation of PRC2 involvement in environmental responses needs to be determined. Interestingly, orthologous genes involved in metabolism and stress response in Brassicaceae species separated by limited evolutionary distance display concordant H3K27me3 marking [161], suggesting conservation of PRC2 involvement in transcriptional regulation of these genes. On the other hand, orthologs involved in developmental functions display higher concordance of H3K27me3 marking in species separated by larger evolutionary distances [161]. This may suggest that developmental modulation by PRC2 is more conserved in evolution, at least within species of certain (limited) evolutionary distance, or that it is less dynamic compared to environmental response regulation and therefore more easily detected.

## 4. PRC2 Targeting to Repeats and Regions of Constitutive Heterochromatin

In mammals and flowering plants in wild-type conditions, H3K27me3 marks chromatin domains of facultative heterochromatin that are distinct from those of constitutive heterochromatin marked by H3K9 methylation (H3K9me) and/or DNA methylation ([154,155,280], reviewed in the works of [9,281]). Increasing evidence nevertheless points to the interplay between mechanisms establishing H3K27me3- and H3K9me/DNAme-marked chromatin in eukaryotes (Table S2) (reviewed in the work of [9,49]). First, H3K27me3 is found to be targeted to TEs in wild-type situations in several unicellular species, including the diatom *Phaeodactylum tricornutum* [165], the red alga *C. merolae* [63], or the ciliate *Paramecium tetraurelia* [106], but also in multicellular species, such as the bryophyte *M. polymorpha* [166] or even *A. thaliana* [282]. Second, in some fungi, H3K27me3 may co-occur with H3K9me [64,238]. Third, H3K27me3 is redistributed to constitutive heterochromatin including TEs upon disruption of H3K9me or DNA hypomethylation in mammals [283,284,285], upon developmental or induced DNA hypomethylation in Arabidopsis [286,287,288,289,290], or upon loss of H3K9me or its reader HETEROCHROMATIN PROTEIN 1 (HP1) in *Neurospora crassa* [291,292] or the pathogenic fungus *Zymoseptoria tritici* [293]. Fourth, disruption of PRC2 can lead to transcriptional activation of TEs in mouse ES cells [294], in ciliates [106,230], or in the green alga *Chlamydomonas reinhardtii* [31]. These pieces of evidence led to the proposal of an ancestral role of PRC2 in repressing transposable elements (reviewed in the work of [49]). However, the contribution of PRC2 to TE silencing may be limited to some TE families [282], as its absence does not aggravate TE de-repression in *A. thaliana* hypomethylated tissues [289,290]. Intriguingly, in *A. thaliana*, the absence of CLF in hypomethylated *ddm1* mutant even promotes DNA re-methylation and chromatin recompaction, suggesting a more intricate interplay between the two silencing pathways [276].

In addition to H3K9me/DNA methylation-associated constitutive heterochromatic regions, emerging reports describe targeting of H3K27me3 to telomeric repeats in different genera and even different eukaryotic supergroups. H3K27me3 is frequently enriched in telomeric and subtelomeric regions in ascomycete and basidiomycete fungi (reviewed in the work of [68]) and contributes to telomere clustering at the nuclear periphery in *N. crassa* [295]. H3K27me3 has been found in telomeres in mammals [296] as well as plants [297,298]. In human cell lines, PRC2 is recruited to telomeres via interaction with telomeric repeat-containing RNAs (TERRAs) [296], lncRNAs originating from the telomeres that contribute to telomere homeostasis and telomeric heterochromatin formation (reviewed in the work of [299]). Interestingly, PRC2 at telomeres is required for H3K9me3, H3K20me3, and HP1 binding [296], suggesting an instrumental role of PRC2 in the establishment of heterochromatin at human telomeres. Noncoding telomeric RNAs are also generated from the (sub)telomeric regions in plants [297,300], and it will be interesting to determine their potential contribution to PRC2 recruitment in plants. In addition to telomeric PRC2 targeting, TERRAs together with TRF1 (telomeric repeat-binding factor 1), a component of the telomere protective complex shelterin (reviewed in the work of [301]), target H3K27me3 to the vicinity of genes involved in pluripotency and developmental control, suggesting conservation of these recruitment mechanisms outside of telomeres [302]. Similarly, in *A. thaliana,* the plant telomere-associated protective components TELOMERE REPEAT BINDING proteins (TRB´s) ([303], reviewed in the work of [304]) interact with PRC2 subunits CLF and SWN, recruiting PRC2 to telobox motifs of H3K27me3-targeted genes [144,305]. Telobox is independently identified among motifs that mediate PRC2 targeting in *A. thaliana* [185], suggesting a more general role of telomeric repeats in genome-wide recruitment of plant PRC2. In *N. crassa*, interstitial telomeric sequence motifs can also initiate ectopic H3K27me3 recruitment and spread into broad domains surrounding the recruitment site [306], suggesting the instructive role of telomeric repeats in PRC2 targeting also in fungi. Targeting of PRC2 to telomeric repeats therefore appears to be conserved in evolution. As TEs that are targeted by PRC2 in the absence of DNA methylation also frequently contain telobox motifs [290], it remains a question whether PRC2 recruitment to telobox motifs in genes is a remnant of ancestral TE targeting or whether ancestral telobox targeting contributed to recruitment to genes and TEs.

## 5. Emerging Patterns and Questions in PRC2 Evolution

Recent work has demonstrated the early emergence of PRC2, its presence, and its fundamental functions in unicellular as well as multicellular eukaryotes, but also secondary loss in some species. Mechanisms that contribute to the dispensability of PRC2 in these species will be interesting to elucidate. The core composition of PRC2 seems to be well conserved in eukaryotic evolution, mainly involving E(z), Esc, and p55, while Su(z)12 function may be dispensable or substituted by other proteins in some species. Compositional diversity may therefore underlie the functional conservation of PRC2. Although PRC2 subunits are predicted to be encoded in the genomes of numerous eukaryotic species, biochemical evidence for their presence and function is lacking. Accessory subunits are known to influence PRC2 recruitment or function and even define different PRC2 complexes, but information about them in different species is very limited. Similarly, only partial understanding of the functional crosstalk between PRC2 and PRC1 exists outside of mammalian models, despite the vital contribution of PRC1 to PRC2 repression. Continued efforts in elucidating aspects of PRC2 composition, subunit interaction, catalytical function, genomic targeting, and dynamics of polycomb-modified chromatin in different species will be crucial for understanding its evolution.

Increasing evidence suggests the ancestral function of PRC2 in facultative heterochromatin organization [49]. A pattern seems to be emerging of H3K27me3 targeting repetitive genomic elements in simple eukaryotes, while gene-specific targeting seems to prevail in complex eukaryotic species. However, how the H3K27me3-catalytic function of PRC2 within facultative heterochromatin in genic regions evolved remains unknown. Several non-exclusive mechanisms can be envisaged, including changes in PRC2 catalytic activity or its genomic targeting. Dual H3K9me and H3K27me activities of E(z)-like proteins in ciliates [106], DNA methylation and/or H3K9me preventing H3K27me3 targeting to TEs in various eukaryotes [49] and recently reported prevention of H3K27me3 targeting to telomeric sequences by histone H1 in *A. thaliana* [307] may provide important hints.

The conserved function of PRC2 in cell identity specification and developmental phase transitions has been established in multicellular model species from different eukaryotic supergroups. PRC2 requirement for morphotype specification in the diatom *P. tricornutum* suggests that its role in cell identity specification may be conserved also in unicellular species [103]. Emerging reports associate PRC2 function with environmental response in multicellular species [5,308] and also with responses to nutrition availability in diverse protists [103]. PRC2 function in dynamic environmental and metabolic responses may therefore be conserved, but further work is needed to determine the involvement of PRC2 subcomplexes and their mode of operation.

Despite recent advances, reports on the composition, biochemical activity, and targeting of PRC2 and on consequences of its dysfunction in unicellular and simple multicellular species are scarce and will be needed to allow more general conclusions as to the evolution of PRC2. PRC2 evolution must be explored in early eukaryotic and monophyletic lineages, including organisms with increasing body plan complexity. Although the green eukaryotic lineage provides a suitable model system for studying PRC2 evolution, our knowledge of PRC2 outside of angiosperms is limited. Recent studies have illuminated exciting differences in H3K27me3 targeting within the bryophyte basal land plant models, but currently, very little is known about PRC2 in green algae. Due to the limited genome availability, PRC2 has been studied only in several chlorophyte algae genera (*Chlamydomonas*, *Volvox,* and *Ostreococcus),* but no streptophyte algae, and further work will be needed to bring more resolution into the PRC2 subunit phylogeny reconstruction. Based on the phylogenetic separation of the PRC2 subunit homologs in the green algae and land plants and the potential loss of Su(z)12 in some of the species [31,89], it will be interesting to determine the variations in the complex characteristics throughout the green lineage.

## Figures and Tables

**Table 1:** The distribution of PRC2 core components in different species (more details in the Supplementary Table S1). (Black **✓** indicates prediction and green **✓** indicates experimental evidence; numbers in brackets indicate the number of homologs identified, ‘?’ indicates that the presence or absence is still unclear, and ‘-’ indicates absence.)

Supergroups	Eukaryotic Group/Kingdom	Phylum or Class	Species	E(z)	Su(z)12	Esc	p55	References
Archaeplastida	Eukaryota	Rhodophyta	*Cyanidioschyzon merolae*	✓	✓	✓	✓ (2)	[63]
	Viridiplantae	Chlorophyta	*Ostreococcus lucimarinus*	✓	✓	✓	✓(2)	[89]
			*Chlamydomonas reinhardtii*	✓	✓	✓(2)	✓(2)	[63,89]
			*Volvox carteri*	✓(2)	?	✓	✓(2)	[89]
	Viridiplantae—Embryophyta	Bryophyta-Bryopsida	*Physcomitrium patens*	✓	✓(3)	✓	✓(2)	[61,62,89,90]
		Lycophyte-Lycopodiopsida	*Selaginella moellendorffii*	✓(2)	✓	✓	✓(3)	[89,90,91]
		Gymnosperm	*Picea abies*	✓	?	?	?	[92]
		Angiosperms-Monocot	*Oryza sativa*	✓ (2)	✓ (2)	✓ (2)	✓ (2)	[91,93,94,95,96]
			*Triticum aestivum*	✓(9)	✓(8)	✓ (7)	✓(6)	[97]
			*Zea mays*	✓ (3)	✓ (2)	✓ (2)	✓ (5)	[94]
			*Brachypodium distachyon*	✓(2)	✓(2)	✓(3)	✓(4)	[89,98,99]
			*Hordeum vulgare*	✓(3)	✓(3)	✓	✓(2)	[94,100,101]
			*Sorghum bicolor*	✓(2)	✓(3)	✓(2)	✓(2)	[94]
		Angiosperms-Eudicot	*Arabidopsis thaliana*	✓ (3)	✓ (3)	✓	✓ (5)	[102]
Chromalveolata	SAR—Stramenopiles	Bacillariophyceae (diatoms)	*Phaeodactylum tricornutum*	✓	✓	✓	✓	[89,103]
		Ochrophyta-Phaeophyceae	*Ectocarpus*	-	-	-	-	[104]
	SAR—Alveolata	Cilliophora/cilliates	*Paramecium tetraurelia*	✓ (2)	-	-	✓	[31,105,106]
			*Tetrahymena thermophila*	✓	✓	✓	✓	[107,108]
Opisthokonta	Fungi	Basidiomycota	*Cryptococcus neoformans*	✓	-	✓	✓	[64,109]
		Ascomycota	*Fusarium graminearum*	✓	✓	✓	✓	[109,110]
			*Chaetomium thermophilum*	✓	✓	✓	✓	[79]
			*Neurospora crassa*	✓	✓	✓	✓	[109,111,112]
			*Saccharomyces cerevisiae*	-	-	-	-	[31,75]
	Filasterea—single-celled eukaryote	Capsaspora	*Capsaspora owczarzaki*	-	-	-	-	[113]
	Animalia/animals	Porifera	*Amphimedon queenslandica*	✓(4)	✓	✓(2)	✓	[114]
		Cnidaria/Hydrozoa	*Hydra vulgaris*	✓	✓	✓	✓	[115,116,117,118]
		Insecta/insects	*Drosophila melanogaster*	✓	✓	✓	✓	[119,120,121]
		Nematoda/nematodes	*Caenorhabditis elegans*	✓	-	✓	-	[122,123]
		Reptilia/reptiles	*Anolis carolinensis*	✓	✓	✓	✓	[124,125,126,127]
		Mammalia/mammals	*Homo sapiens*	✓ (2)	✓	✓	✓	[56,60]
			*Mus musculus*	✓ (2)	✓	✓	✓	[128,129]

## Data Availability

Not applicable.

## Supplementary Materials

Supplementary Tables are available online at https://www.mdpi.com/article/10.3390/epigenomes6010003/s1, Table S1: PRC2 core components in different species, Table S2: A general overview of the distribution of histone methylation marks (H3K27me3) and its putative functions across various species.
