# Supplementary material for: Polycomb Repressive Complex 2 in Eukaryotes—An Evolutionary Perspective
Source: Epigenomes. 2022 Jan 17;6(1):3. doi: 10.3390/epigenomes6010003 (PMC8788455; doi:10.3390/epigenomes6010003)
Supplement: Supplementary file 1 [file epigenomes-06-00003-s001.zip › epigenomes-1502141-supplementary.pdf]

Table S1. **PRC2 core components in different species**

(Abbreviations: E(z) - Enhancer of zeste, Su(z)12 - Suppressor of zeste 12, ESC - Extra sex combs, Nurf55 - Nucleosome remodelling factor (also called p55), MSI - Multicopy Suppressor of IRA, MEZ - Maize Enhancer of zeste-like, CLF - CURLY LEAF, SWN - SWINGER, MEA -MEDEA, EMF2 - EMBRYONIC FLOWER 2, FIS2 - FERTILIZATION INDEPENDENT SEED 2, VRN2 - VERNALIZATION 2, FIE- FERTILIZATION INDEPENDENT ENDOSPERM, ESCL - ESC-like, MES- Maternal-Effect sterile, RBBP- Retinoblastoma Binding Protein, RbAp-Retinoblastoma Associated protein CAF1 - Chromatin Assembly Factor 1, NPF - *Neurospora* homolog of *Drosophila* P55 and mammalian P48.). Percentage identity/similarity (based on EMBOSS Needle[1]) to the *Drosophila melanogaster* PRC2 subunits are given in brackets (only full-length sequences available in the NCBI GenBank were used).

Supergroups	Eukaryotic group/Kingdom	Phylum or Class	Species	E(z) homolog	Su(z) homolog	Esc homolog	p55 homolog	References
Archaeplastida	Eukaryota	Rhodophyta	*Cyanidioschyzon merolae*	CmE(z)<br>(24.3/ 37.6)	CmSu(z)12	CmESC	p55 (2)<br>(46.3/63.0)	[2]
	Viridiplantae	Chlorophyta	*Ostreococcus lucimarinus*	OlKMT6A2<br>(20.1/29.8)	OlEMF2	OlFIE	OlMSI1, OlMSI4<br>(59.1/73.0)	[3]
			Chlamydomonas reinhardtii	CrE(z)/CrKMT6A2<br>(22.2/33.0)	CrEMF2<br>(12.1/21.5)	CrESC.1/2 or CrFIE<br>(26.6/39.9)	CrMSI1 (59.6/74.5)<br>CrMSI4	[2,3]
			Volvox carteri	VcKMT6A1;<br>VcKMT6A3	-	VcFIE	VcMSI1 (61.5/74.9)<br>VcMSI4	[3]
	Viridiplantae - Embryophyta	Bryophyta - Bryopsida	*Physcomitrium patens (Moss)*	PpCLF<br>(22.5/31.1)	PpEMF2a,<br>PpEMF2b,<br>PpEMF2c<br>(13.1/22.4)	PpFIE<br>(31.9/50.0)	PpMSI1 (64.7/79.8),<br>PpMSI4	[3–6]
		Lycophyte - Lycopodiopsida	*Selaginella moellendorffii* (primitive spikemoss)	SmKMT6A 1a<br>SmKMT6A 1b<br>(CLF like)<br>(21.9/32.6)	SdEMF2p,<br>SdEMF2p_1<br>(13.6/22.7)	SmFIE<br>(34.5/52.1)	SmMSI1 (62.3/74.9)<br>SmMSI2a, SmMSI2b	[3,6,7]
		Gymnosperm	*Picea abies*	E(z) (CLF)	?	?	?	[8]
		Angiosperms - Monocot	*Oryza sativa (Rice)*	OsiEZ1 (OsSWN)<br>(21.3/32.6),<br>OsCLF (23.8/36.4)	OsEMF2a,<br>OsEMF2b<br>(13.6/24.0)	OsFIE1<br>(32.5/47.8),<br>OsFIE2	OsMSI1 (62.4/76.8),<br>OsMSI2,	[7,9–12]
			Triticum aestivum (Bread wheat)	TaE(z)-4A1,<br>TaE(z)-7A1.1,<br>TaE(z)-7A1.2<br>(24.4/36.9)	TaSu(z)-2A1,<br>TaSu(z)-2A2,<br>TaSu(z)-5A1<br>(16.0/27.8)	TaFIE-7A1,<br>TaFIE-7A2.1,<br>TaFIE-7A2.2,<br>TaFIE-4A1<br>(34.0/51.9)	TaMSI1-A1,<br>TaMSI1-A2<br>(62.4/77.0)	[13]
			Zea mays (Maize)	ZmMEZ1 (CLF)<br>(22.2/34.8),<br>ZmMEZ2 (SWN1)	ZmEMF2a/2-1,<br>ZmEMF2b/2-2<br>(15.1/26.8)	ZmFIE1, ZmFIE2<br>(33.7/49.2)	ZmMSI1-1<br>(62.4/77.4),<br>ZmMSI1-2,	[10]

				(24.1/36.9), ZmMEZ3 (SWN2)			ZmMSI1-3, ZmMSI1-4, ZmMSI2	
			Brachypodium distachyon (Model grass)	BdEZL1 (CLF ortholog, BdCLF) (27.6/41.6), BdEZL2 (SWN ortholog, BdSWN)	BdEMF2a, BdEMF2b (14.8/23.6)	BdFIE1, bdFIE2, bdFIE3 (33.8/51.2)	BdMSI1a (62.1/77.4), BdMSI1b, BdMSI3, BdMSI4	[3,14,15]
			Hordeum vulgare (Barley)	HvCLF1, HvCLF2, HvSWN (25.6/39.4)	HvSu(z)12a (HvEMF2a), HvSu(z)12b (HvEMF2b), HvSu(z)12c (HvEMF2c) (15.6/27.5)	HvFIE (26.8/41.5)	HvMSI1 (62.4/76.8), HvMSI2	[10,16,17]
			Sorghum bicolor	SbCLF (20.6/32.2), SbSWN	SbEMF2a (15.2/25.3), SbEMF2b, SbEMF2c	SbFIE1, SbFIE2 (28.2/43.3)	SbMSI1 (63.0/77.4), SbMSI2	[10]
		Angiosperms - Eudicot	*Arabidopsis thaliana*	CLF (23.7/36.6), SWN (24.8/37.4), MEA (26.0/38.2)	EMF2 (15.0/26.8), FIS2 (14.4/25.0), VRN2 (10.5/17.9)	FIE (33.3/50.5)	MSI1 (62.9/77.3), MSI2, MSI3, MSI4, MSI5	[18]
Chromalveolata	SAR-Stramenopiles	Bacillariophyceae (diatoms)	*Phaeodactylum tricornutum*	Ez	Suz12	Esc	Nurf55(*p55*) (48.2/61.9)	[3,19]
	SAR - Alveolata	Cilliophora/ cilliates	*Paramecium tetraurelia*	Ezl1p (20.3/36.0), Ezl2p	-	_	Nurf55 (47.0/62.8)	[20–22]
			Tetrahymena thermophila	EZL1 (E(z)-like 1) (18.5/31.0)	SUZ12	ESC1	RebL1 (47.5/65.1)	[23,24]
Opisthokonta	Fungi	Basidiomycota	*Cryptococcus neoformans*	Ezh2 (20.8/30.8)	-	Eed1 (20.5/31.3)	Msl1 (51.5/67.7)	[25,26]
		Ascomycota	*Fusarium graminearum/ Gibberella zeae*	SET-7/KMT6 (13.4/22.3)	Suz12	EED (19.5/31.0)	MSL1 (43.9/62.1), CAF1-3	[26,27]
			Chaetomium thermophilum	Ezh2 (11.3/19.2)	Su(z)12	EED (17.0/27.4)	p55 (45.4/66.5)	[28,29]
			Neurospora crassa	SET-7 (KMT6) (11.7/18.9)	SUZ12 (15.0/25.1)	EED (16.2/25.6)	NPF (p55) (44.8/64.4)	[26,30,31]
			Saccharomyces cerevisiae	-	-	-	-	[22,32]
	Filasterea- single-celled eukaryote	Capsaspora	*Capsaspora owczarzaki*	-	-	-	-	[33]
	Animalia/animals	Porifera	*Amphimedon queenslandica*	E(z) (4 copies)	Su(z)12 (17.0/24.7)	ESC, ESCL	Nurf55	[34]
		Cnidaria/ Hydrozoa	*Hydra vulgaris* (swiftwater hydra)	HyEZH2	Su(z)12-like (predicted) (20.5/35.3)	HyEED (42.7/62.1)	RBBP7	[35–38]

		Insecta/ insects	*Drosophila melanogaster*	E(z) (100/100)	Su(z)12 (100/100)	Esc/Escl (100/100)	p55 (Nurf55) (100/100)	[39–41]
		Nematoda/ nematodes	*Caenorhabditis elegans*	MES-2 (22.6/33.5)	-	MES-6 (23.4/36.9)	-	[42,43]
		Reptilia/ reptiles	*Anolis carolinensis (green anole/chameleon)*	AcEZH2 (52.1/62.2)	SUZ12 (26.1/38.2)	EED (46.4/63.9)	RBBP4	[44–47]
		Mammalia/ mammals	*Homo sapiens*	EZH1(50.3/63.2), EZH2 (53.5/63.8)	SUZ12 (28.8/42.6)	EED (47.8/66.6)	RBBP4/7 (RbAp48 /RbAp46) (87.8/93.8)	[48,49]
			Mus musculus (Mouse)	EZH1(49.9/62.9), EZH2 (54.3/64.4)	SUZ12 (28.3/42.0)	EED (48.2/66.7)	RbAp48 (87.5/93.5)	[50,51]

Table S2. A general overview at the distribution of H3K27me3 and its putative functions across various studied species.

**Supergroups**	**Eukaryotic group/ Kingdom**	**Phylum or Class**	**Species**	**H3K27me3 distribution**	**H3K27me3 distribution related to H3K9me2/3 and/or DNA methylation**	**Proposed function**	**References**
Archaeplastida	Eukaryota	Rhodophyta	*Cyanidioschyzon merolae*	Telomeric and subtelomeric regions (4% annotated genes and 50 % repetitive elements)	Unknown	Regulate gene repression especially associated with Intein-mediated splicing of proteins.	[2]
	Viridiplantae	Chlorophyta	*Chlamydomonas reinhardtii*	H3K27me3 absent or present in very low levels	No effect on K9me1/3 or DNA methylation	Repression of repetitive genomic elements	[22]
	Viridiplantae - Embryophyta	Bryophyta - Bryopsida	*Physcomitrium patens*	Genes (preference for shorter genes)	Non-overlapping	Repression of apogamy	[4,5,52]
		Marchantiophyta-Marchantiopsida	*Marchantia polymorpha*	10% annotated genes and 20 % in repetitive elements	Non-overlapping	Repression of genes and repeats (TEs)	[53]
		Pinophyta – Pinopsida (Gymnosperm)	*Picea abies*	Genes-uniformly distributed, additionally clustered in the mid arm position	Unknown	Embryogenesis, cell-fate determination	[8,54]
			Pinus sylvestris		Unknown		
		Angiosperms -Eudicot	*Arabidopsis thaliana*	20% – 30% annotated genes (domains <1kb)	Mutually exclusive, H3K27me3 spreading into H3K9me2-regions upon DNA demethylation	Development, phase transitions	[55–62]

Chromalveolata	SAR-Stramenopiles	Bacillariophyceae (diatoms)	*Phaeodactylum tricornutum*	7% annotated genes, 41% annotated TE and intergenic region	H3K27me3, H3K9me2/3, and DNA methylation coexist (H3K27me3 overlap with H3K9me2)	Transcriptional silencing (role in signal transduction, development and cell cycle control; morphology/cell differentiation)	[19,63]
		Ochrophyta-Phaeophyceae	*Ectocarpus*	H3K27me3 present but distribution unknown	Unknown	Unknown	[64]
	SAR - Alveolata	Cilliophora/cilliates (Chromalveolata)	*Tetrahymena thermophila*	micronucleus (K27&K9me1/2/3); macronucleus especially anlagen	Co-exist	Developmental regulation and foreign DNA elimination during somatic differentiation, macronucleus differentiation	[65,66]
			Paramecium tetraurelia	Newly developing macronucleus	Co-exist- H3K27me3 and H3K9me3 co-occur at TEs	Control RNAi, Transcriptional silencing of transposable elements (for the development of sexually viable progeny and the elimination of DNA)	[21]
Opisthokonta	Fungi	Basidiomycota	*Cryptococcus neoformans*	Subtelomeres - broad domains (average 41 kb). On repetitive elements cover 5% of the genome	Redistribution to centromeric H3K9me2-marked regions upon disruption of H3K27me3-marked chromatin binding; redistribution depends on H3K9me2 deposition	Dispensable for normal growth and development	[25]
		Ascomycota	*Neurospora crassa*	Subtelomeres and telomeres, including genes (broad domain- ~12.5 kb)	H3K27me3 and H3K9me3 form distinct domains at telomeric regions (adjacent, but no overlap)	Developmental regulations	[30,31,67]
			Saccharomyces cerevisiae and *S. pombe*	Lacks H3K27 methylation	-	-	[68]
			Candida albicans	Lacks H3K27 methylation	-	-	[68]
	Animalia/animals	Porifera	*Amphimedon queenslandica*	Gene bodies	Unknown	Developmental regulatory genes	[34]
		Insecta/insects	*Drosophila melanogaster*	Genes (broad domains ~70kb)	H3K27me3 deposition is independent from DNA methylation.<br><br>Non-overlapping or antagonistic relationship	Gene silencing or facultative heterochromatin formation (Developmental gene regulation)	[69]

					between H3K27me3 and H3K36me2/3 (De et al., 2020)		
		Nematoda/nematodes	*Caenorhabditis elegans*	X-chromosome, H3K27me3 levels are higher on arm regions compared to the centre	H3K9me3-marked chromatin coincides with H3K27me3.Antagonistic relationship between H3K27me3 and H3K36me2/3	Germline development, repression of X-chromosome	[43,70–72]
		Mammalia/mammals	Mus musculus, Homo sapiens	Genes, telomeres (Broad domains (~43kb))	H3K27me3 and H3K9me3 distributions are mutually exclusive.	Developmental gene regulation	[73,74]

References

1. EMBOSS Needle. Available Online: Https://Www.Ebi.Ac.Uk/Tools/Psa/Emboss_needle/.
2. Mikulski, P.; Komarynets, O.; Fachinelli, F.; Weber, A.P.M.; Schubert, D. Characterization of the Polycomb-Group Mark H3K27me3 in Unicellular Algae. *Frontiers in Plant Science* **2017**, *8*, doi:10.3389/fpls.2017.00607.
3. Huang, Y.; Chen, D.H.; Liu, B.Y.; Shen, W.H.; Ruan, Y. Conservation and Diversification of Polycomb Repressive Complex 2 (PRC2) Proteins in the Green Lineage. *Brief Funct Genomics* **2016**, doi:10.1093/bfgp/elw007.
4. Okano, Y.; Aono, N.; Hiwatashi, Y.; Murata, T.; Nishiyama, T.; Ishikawa, T.; Kubo, M.; Hasebe, M. A Polycomb Repressive Complex 2 Gene Regulates Apogamy and Gives Evolutionary Insights into Early Land Plant Evolution. *Proceedings of the National Academy of Sciences of the United States of America* **2009**, *106*, 16321–16326, doi:10.1073/pnas.0906997106.
5. Mosquna, A.; Katz, A.; Decker, E.L.; Rensing, S.A.; Reski, R.; Ohad, N. Regulation of Stem Cell Maintenance by the Polycomb Protein FIE Has Been Conserved during Land Plant Evolution. *Development* **2009**, *136*, 2433–2444, doi:10.1242/dev.035048.
6. Chen, L.J.; Diao, Z.Y.; Specht, C.; Sung, Z.R. Molecular Evolution of VEF-Domain-Containing PcG Genes in Plants. *Molecular Plant* **2009**, *2*, 738–754, doi:10.1093/mp/ssp032.
7. Luo, M.; Platten, D.; Chaudhury, A.; Peacock, W.J.; Dennis, E.S. Expression, Imprinting, and Evolution of Rice Homologs of the Polycomb Group Genes. *Molecular Plant* **2009**, *2*, 711–723, doi:10.1093/mp/ssp036.
8. Nakamura, M.; Batista, R.A.; Köhler, C.; Hennig, L. Polycomb Repressive Complex 2-Mediated Histone Modification H3K27me3 Is Associated with Embryogenic Potential in Norway Spruce. *Journal of Experimental Botany* **2021**, *71*, 6366–6378, doi:10.1093/JXB/ERAA365.
9. Liu, X.; Zhou, C.; Zhao, Y.; Zhou, S.; Wang, W.; Zhou, D.X. The Rice Enhancer of Zeste [E(z)] Genes SDG711 and SDG718 Are Respectively Involved in Long Day and Short Day Signaling to Mediate the Accurate Photoperiod Control of Flowering Time. *Frontiers in Plant Science* **2014**, *5*, 1–9, doi:10.3389/fpls.2014.00591.
10. Ni, J.; Ma, X.; Feng, Y.; Tian, Q.; Wang, Y.; Xu, N.; Tang, J.; Wang, G. Updating and Interaction of Polycomb Repressive Complex 2 Components in Maize (Zea Mays). *Planta* **2019**, *250*, doi:10.1007/s00425-019-03193-4.
11. Conrad, L.J.; Khanday, I.; Johnson, C.; Guiderdoni, E.; An, G.; Vijayraghavan, U.; Sundaresan, V. The Polycomb Group Gene EMF2B Is Essential for Maintenance of Floral Meristem Determinacy in Rice. *The Plant Journal* **2014**, *80*, 883–894, doi:10.1111/tpj.12688.

12. Cheng, X.; Pan, M.; E, Z.; Zhou, Y.; Niu, B.; Chen, C. The Maternally Expressed Polycomb Group Gene OsEMF2a Is Essential for Endosperm Cellularization and Imprinting in Rice. *Plant Commun* **2020**, *2*, 100092–100092, doi:10.1016/j.xplc.2020.100092.
13. Strejčková, B.; Čegan, R.; Pecinka, A.; Milec, Z.; Šafář, J. Identification of Polycomb Repressive Complex 1 and 2 Core Components in Hexaploid Bread Wheat. *BMC Plant Biology* **2020**, *20*, 1–13, doi:10.1186/s12870-020-02384-6.
14. Higgins, J.A.; Bailey, P.C.; Laurie, D.A. Comparative Genomics of Flowering Time Pathways Using Brachypodium Distachyon as a Model for the Temperate Grasses. *PLoS ONE* **2010**, *5*, doi:10.1371/journal.pone.0010065.
15. Lomax, A.; Woods, D.P.; Dong, Y.; Bouché, F.; Rong, Y.; Mayer, K.S.; Zhong, X.; Amasino, R.M. An Ortholog of CURLY LEAF/ENHANCER OF ZESTE like-1 Is Required for Proper Flowering in Brachypodium Distachyon. *The Plant Journal* **2018**, *93*, 871–882, doi:10.1111/tpj.13815.
16. Kapazoglou, A.; Tondelli, A.; Papaefthimiou, D.; Ampatzidou, H.; Francia, E.; Stanca, M.A.; Bladenopoulos, K.; Tsaftaris, A.S. Epigenetic Chromatin Modifiers in Barley: IV. The Study of Barley Polycomb Group (PcG) Genes during Seed Development and in Response to External ABA. *BMC Plant Biology* **2010**, *10*, doi:10.1186/1471-2229-10-73.
17. Tonosaki, K.; Kinoshita, T. Possible Roles for Polycomb Repressive Complex 2 in Cereal Endosperm. *Frontiers in Plant Science* **2015**, *6*, 1–5, doi:10.3389/fpls.2015.00144.
18. Derkacheva, M.; Steinbach, Y.; Wildhaber, T.; Mozgová, I.; Mahrez, W.; Nanni, P.; Bischof, S.; Gruissem, W.; Hennig, L. Arabidopsis MSI1 Connects LHP1 to PRC2 Complexes. *EMBO Journal* **2013**, *32*, 2073–2085, doi:10.1038/emboj.2013.145.
19. Zhao, X.; Deton Cabanillas, A.F.; Veluchamy, A.; Bowler, C.; Vieira, F.R.J.; Tirichine, L. Probing the Diversity of Polycomb and Trithorax Proteins in Cultured and Environmentally Sampled Microalgae. *Frontiers in Marine Science* **2020**, *7*, 1–17, doi:10.3389/fmars.2020.00189.
20. Rzeszutek, I.; Maurer-Alcalá, X.X.; Nowacki, M. Programmed Genome Rearrangements in Ciliates. *Cellular and Molecular Life Sciences* **2020**, *77*, 4615–4629, doi:10.1007/s00018-020-03555-2.
21. Frapporti, A.; Miró Pina, C.; Arnaiz, O.; Holoch, D.; Kawaguchi, T.; Humbert, A.; Eleftheriou, E.; Lombard, B.; Loew, D.; Sperling, L.; et al. The Polycomb Protein Ezl1 Mediates H3K9 and H3K27 Methylation to Repress Transposable Elements in Paramecium. *Nature Communications* **2019**, *10*, 2710, doi:10.1038/s41467-019-10648-5.
22. Shaver, S.; Casas-Mollano, J.A.; Cerny, R.L.; Cerutti, H. Origin of the Polycomb Repressive Complex 2 and Gene Silencing by an E(z) Homolog in the Unicellular Alga Chlamydomonas. *Epigenetics* **2010**, *5*, 301–312.
23. Xu, J.; Zhao, X.; Mao, F.; Basrur, V.; Ueberheide, B.; Chait, B.T.; David Allis, C.; Taverna, S.D.; Gao, S.; Wang, W.; et al. A Polycomb Repressive Complex Is Required for RNAi-Mediated Heterochromatin Formation and Dynamic Distribution of Nuclear Bodies. *Nucleic Acids Research* **2021**, *49*, 5407–5425, doi:10.1093/nar/gkaa1262.
24. Nabeel-Shah, S.; Garg, J.; Saettone, A.; Ashraf, K.; Lee, H.; Wahab, S.; Ahmed, N.; Fine, J.; Derynck, J.; Pu, S.; et al. Functional Characterization of RebL1 Highlights the Evolutionary Conservation of Oncogenic Activities of the RBBP4/7 Orthologue in Tetrahymena Thermophila. *Nucleic Acids Research* **2021**, *49*, 6196–6212, doi:10.1093/nar/gkab413.
25. Dumesic, P.A.; Homer, C.M.; Moresco, J.J.; Pack, L.R.; Shanle, E.K.; Coyle, S.M.; Strahl, B.D.; Fujimori, D.G.; Yates, J.R.; Madhani, H.D. Product Binding Enforces the Genomic Specificity of a Yeast Polycomb Repressive Complex. *Cell* **2015**, *160*, 204–218, doi:10.1016/j.cell.2014.11.039.
26. Ridenour, J.B.; Möller, M.; Freitag, M. Polycomb Repression without Bristles: Facultative Heterochromatin and Genome Stability in Fungi. *Genes* 2020, *11*.
27. Connolly, L.R.; Smith, K.M.; Freitag, M. The Fusarium Graminearum Histone H3 K27 Methyltransferase KMT6 Regulates Development and Expression of Secondary Metabolite Gene Clusters. *PLoS Genetics* **2013**, *9*, doi:10.1371/journal.pgen.1003916.
28. Jiao, L.; Liu, X. Structural Analysis of an Active Fungal PRC2. *Nucleus* **2016**, *7*, 284–291, doi:10.1080/19491034.2016.1183849.
29. Jiao, L.; Liu, X. Structural Basis of Histone H3K27 Trimethylation by an Active Polycomb Repressive Complex 2. *Science* **2015**, *350*, doi:10.1126/science.aac4383.

30. Jamieson, K.; Rountree, M.R.; Lewis, Z.A.; Stajich, J.E.; Selker, E.U. Regional Control of Histone H3 Lysine 27 Methylation in Neurospora. *Proc Natl Acad Sci U S A* **2013**, *110*, 6027–6032, doi:10.1073/pnas.1303750110.
31. McNaught, K.J.; Wiles, E.T.; Selker, E.U. Identification of a PRC2 Accessory Subunit Required for Subtelomeric H3K27 Methylation in Neurospora Crassa. *Molecular and Cellular Biology* **2020**, *40*, doi:10.1128/MCB.00003-20.
32. Margueron, R.; Reinberg, D. The Polycomb Complex PRC2 and Its Mark in Life. *Nature* 2011, *469*, 343–349.
33. Sebé-Pedrós, A.; Ballaré, C.; Parra-Acero, H.; Chiva, C.; Tena, J.J.; Sabidó, E.; Gómez-Skarmeta, J.L.; Di Croce, L.; Ruiz-Trillo, I. The Dynamic Regulatory Genome of Capsaspora and the Origin of Animal Multicellularity. *Cell* **2016**, *165*, 1224–1237, doi:10.1016/j.cell.2016.03.034.
34. Gaiti, F.; Jindrich, K.; Fernandez-Valverde, S.L.; Roper, K.E.; Degnan, B.M.; Tanurdžić, M. Landscape of Histone Modifications in a Sponge Reveals the Origin of Animal Cis-Regulatory Complexity. *eLife* **2017**, *6*, 1–33, doi:10.7554/eLife.22194.
35. Genikhovich, G.; Kürn, U.; Hemmrich, G.; Bosch, T.C.G. Discovery of Genes Expressed in Hydra Embryogenesis. *Developmental Biology* **2006**, *289*, 466–481, doi:10.1016/j.ydbio.2005.10.028.
36. Pillai, A.; Gungi, A.; Reddy, P.C.; Galande, S. Epigenetic Regulation in Hydra: Conserved and Divergent Roles. *Frontiers in Cell and Developmental Biology* **2021**, *9*, 1–23, doi:10.3389/fcell.2021.663208.
37. *PREDICTED: Polycomb Protein Suz12-like [Hydra Vulgaris]. Available Online: Https://Www.Ncbi.Nlm.Nih.Gov/Protein/XP_012556196.1?Report=genbank&log$=prottop&blast_rank=1&RID=R2KAB9PA013 (Accessed on 21/10/2021).*;
38. PREDICTED: Histone-Binding Protein RBBP7 [Hydra Vulgaris]. Available Online: Https://Www.Ncbi.Nlm.Nih.Gov/Protein/XP_012554465.1?Report=genbank&log$=prottop&blast_rank=1&RID=R2KUGECN013 (Accessed on 21/10/2021).
39. Herz, H.-M.; Mohan, M.; Garrett, A.S.; Miller, C.; Casto, D.; Zhang, Y.; Seidel, C.; Haug, J.S.; Florens, L.; Washburn, M.P.; et al. Polycomb Repressive Complex 2-Dependent and -Independent Functions of Jarid2 in Transcriptional Regulation in Drosophila. *Molecular and Cellular Biology* **2012**, *32*, 1683–1693, doi:10.1128/mcb.06503-11.
40. Schwartz, Y.B.; Pirrotta, V. A New World of Polycombs: Unexpected Partnerships and Emerging Functions. *Nature Reviews Genetics* **2013**, *14*, 853–864, doi:10.1038/nrg3603.
41. Erokhin, M.; Gorbenko, F.; Lomaev, D.; Mazina, M.Y.; Mikhailova, A.; Garaev, A.K.; Parshikov, A.; Vorobyeva, N.E.; Georgiev, P.; Schedl, P.; et al. Boundaries Potentiate Polycomb Response Element-Mediated Silencing. *BMC Biology* **2021**, *19*, 1–20, doi:10.1186/s12915-021-01047-8.
42. Bender LB, Cao R, Zhang Y, S.S. The MES-2/MES-3/MES-6 Complex and Regulation of Histone H3 Methylation in C. Elegans. *Curr Biol.* **2004**, *14*, 1639–1643, doi:10.1016/j.cub.2004.08.062.
43. Gaydos, L.J.; Wang, W.; Strome, S. H3K27me and PRC2 Transmit a Memory of Repression across Generations and during Development. *Science* **2014**, *345*, 1515–1518, doi:10.1126/science.1255023.
44. Brooun, A.; Gajiwala, K.S.; Deng, Y.L.; Liu, W.; Bolaños, B.; Bingham, P.; He, Y.A.; Diehl, W.; Grable, N.; Kung, P.P.; et al. Polycomb Repressive Complex 2 Structure with Inhibitor Reveals a Mechanism of Activation and Drug Resistance. *Nature communications* **2016**, *7*, 11384, doi:10.1038/ncomms11384.
45. PREDICTED: LOW QUALITY PROTEIN: Polycomb Protein SUZ12 [Anolis Carolinensis]. Available Online: Https://Www.Ncbi.Nlm.Nih.Gov/Protein/XP_008112590.1?Report=genbank&log$=prottop&blast_rank=1&RID=R2PMHJ83016 (Accessed on 21/10/2021).
46. PREDICTED: Polycomb Protein EED [Anolis Carolinensis]. Available Online: Https://Www.Ncbi.Nlm.Nih.Gov/Protein/XP_003219406.1?Report=genbank&log$=prottop&blast_rank=1&RID=R2PDKH5B013 (Accessed on 21/10/2021).
47. PREDICTED: Histone-Binding Protein RBBP4 Isoform X2 [Anolis Carolinensis]. Available Online: Https://Www.Ncbi.Nlm.Nih.Gov/Protein/XP_003230003.3?Report=genbank&log$=prottop&blast_rank=1&RID=R2P2UE3N013 (Accessed on 21/10/2021).

48. Kasinath, V.; Faini, M.; Poepsel, S.; Reif, D.; Feng, X.A.; Stjepanovic, G.; Aebersold, R.; Nogales, E. Structures of Human PRC2 with Its Cofactors AEBP2 and JARID2. *Science* **2018**, *359*, doi:10.1126/science.aar5700.
49. Holoch, D.; Margueron, R. Mechanisms Regulating PRC2 Recruitment and Enzymatic Activity. *Trends in Biochemical Sciences* **2017**, *42*, 531–542, doi:10.1016/j.tibs.2017.04.003.
50. Meng, T.G.; Zhou, Q.; Ma, X.S.; Liu, X.Y.; Meng, Q.R.; Huang, X.J.; Liu, H.L.; Lei, W.L.; Zhao, Z.H.; Ouyang, Y.C.; et al. PRC2 and EHMT1 Regulate H3K27me2 and H3K27me3 Establishment across the Zygote Genome. *Nature Communications* **2020**, *11*, 1–12, doi:10.1038/s41467-020-20242-9.
51. He, A.; Ma, Q.; Cao, J.; von Gise, A.; Zhou, P.; Xie, H.; Zhang, B.; Hsing, M.; Christodoulou, D.C.; Cahan, P.; et al. Polycomb Repressive Complex 2 Regulates Normal Development of the Mouse Heart. *Circulation Research* **2012**, *110*, 406–415, doi:10.1161/CIRCRESAHA.111.252205.
52. Widiez, T.; Symeonidi, A.; Luo, C.; Lam, E.; Lawton, M.; Rensing, S.A. The Chromatin Landscape of the Moss Physcomitrella Patens and Its Dynamics during Development and Drought Stress. *The Plant Journal* **2014**, *79*, 67–81, doi:10.1111/tpj.12542.
53. Montgomery, S.A.; Tanizawa, Y.; Galik, B.; Wang, N.; Ito, T.; Mochizuki, T.; Akimcheva, S.; Bowman, J.L.; Cognat, V.; Maréchal-Drouard, L.; et al. Chromatin Organization in Early Land Plants Reveals an Ancestral Association between H3K27me3, Transposons, and Constitutive Heterochromatin. *Current Biology* **2020**, *30*, 573-588.e7, doi:10.1016/j.cub.2019.12.015.
54. Fuchs, J.; Jovtchev, G.; Schubert, I. The Chromosomal Distribution of Histone Methylation Marks in Gymnosperms Differs from That of Angiosperms. *Chromosome Research* **2008**, *16*, 891–898, doi:10.1007/s10577-008-1252-4.
55. Dong, X.; Reimer, J.; Göbel, U.; Engelhorn, J.; He, F.; Schoof, H.; Turck, F. Natural Variation of H3K27me3 Distribution between Two Arabidopsis Accessions and Its Association with Flanking Transposable Elements. *Genome Biology* **2012**, *13*, doi:10.1186/gb-2012-13-12-r117.
56. Turck, F.; Roudier, F.; Farrona, S.; Martin-Magniette, M.L.; Guillaume, E.; Buisine, N.; Gagnot, S.; Martienssen, R.A.; Coupland, G.; Colot, V. Arabidopsis TFL2/LHP1 Specifically Associates with Genes Marked by Trimethylation of Histone H3 Lysine 27. *PLoS Genetics* **2007**, *3*, 0855–0866, doi:10.1371/journal.pgen.0030086.
57. Deleris, A.; Stroud, H.; Bernatavichute, Y.; Johnson, E.; Klein, G.; Schubert, D.; Jacobsen, S.E. Loss of the DNA Methyltransferase MET1 Induces H3K9 Hypermethylation at PcG Target Genes and Redistribution of H3K27 Trimethylation to Transposons in Arabidopsis Thaliana. *PLoS Genetics* **2012**, *8*, doi:10.1371/journal.pgen.1003062.
58. Weinhofer, I.; Hehenberger, E.; Roszak, P.; Hennig, L.; Köhler, C. H3K27me3 Profiling of the Endosperm Implies Exclusion of Polycomb Group Protein Targeting by DNA Methylation. *PLoS Genetics* **2010**, *6*, 1–14, doi:10.1371/journal.pgen.1001152.
59. Moreno-Romero, J.; Jiang, H.; Santos-González, J.; Köhler, C. Parental Epigenetic Asymmetry of PRC 2-mediated Histone Modifications in the Arabidopsis Endosperm. *The EMBO Journal* **2016**, *35*, 1298–1311, doi:10.15252/embj.201593534.
60. Schubert, D. Evolution of Polycomb-Group Function in the Green Lineage. *F1000Research* **2019**, *8*, 268, doi:10.12688/f1000research.16986.1.
61. Zervudacki, J.; Yu, A.; Amesefe, D.; Wang, J.; Drouaud, J.; Navarro, L.; Deleris, A. Transcriptional Control and Exploitation of an Immune-Responsive Family of Plant Retrotransposons. *EMBO J* **2018**, *37*, e98482, doi:10.15252/embj.201798482.
62. Vaquero-Sedas, M.I.; Vega-Palas, M.A. Differential Association of Arabidopsis Telomeres and Centromeres with Histone H3 Variants. *Scientific Reports* **2013**, *3*, 1202, doi:10.1038/srep01202.
63. Veluchamy, A.; Rastogi, A.; Lin, X.; Lombard, B.; Murik, O.; Thomas, Y.; Dingli, F.; Rivarola, M.; Ott, S.; Liu, X.; et al. An Integrative Analysis of Post-Translational Histone Modifications in the Marine Diatom Phaeodactylum Tricornutum. *Genome Biology* **2015**, *16*, 102, doi:10.1186/s13059-015-0671-8.
64. Bourdareau, S.; Tirichine, L.; Lombard, B.; Loew, D.; Scornet, D.; Wu, Y.; Coelho, S.M.; Cock, J.M. Histone Modifications during the Life Cycle of the Brown Alga Ectocarpus. *Genome Biology* **2021**, *22*, 1–27, doi:10.1186/s13059-020-02216-8.
65. Liu, Y.; Taverna, S.D.; Muratore, T.L.; Shabanowitz, J.; Hunt, D.F.; Allis, C.D. RNAi-Dependent H3K27 Methylation Is Required for Heterochromatin Formation and DNA Elimination in Tetrahymena. *Genes and Development* **2007**, *21*, 1530–1545, doi:10.1101/gad.1544207.

66. Chung, P.H.; Yao, M.C. Tetrahymena Thermophila JMJD3 Homolog Regulates H3K27 Methylation and Nuclear Differentiation. *Eukaryotic Cell* **2012**, *11*, 601–614, doi:10.1128/EC.05290-11.
67. Jamieson, K.; McNaught, K.J.; Ormsby, T.; Leggett, N.A.; Honda, S.; Selker, E.U. Telomere Repeats Induce Domains of H3K27 Methylation in Neurospora. *eLife* **2018**, *7*, 1–18, doi:10.7554/eLife.31216.
68. Freitag, M. Histone Methylation by SET Domain Proteins in Fungi. *Annual Review of Microbiology* 2017, *71*.
69. De, S.; Gehred, N.D.; Fujioka, M.; Chan, F.W.; Jaynes, J.B.; Kassis, J.A. Defining the Boundaries of Polycomb Domains in Drosophila. *Genetics* **2020**, *216*, 689–700, doi:10.1534/genetics.120.303642.
70. Ahringer, J.; Gasser, S.M. Repressive Chromatin in Caenorhabditis Elegans: Establishment, Composition, and Function. *Genetics* **2018**, *208*, 491–511, doi:10.1534/genetics.117.300386.
71. Gaydos, L.; Rechtsteiner, A.; Egelhofer, T.; Carroll, C.; Strome, S. Antagonism between MES-4 and Polycomb Repressive Complex 2 Promotes Appropriate Gene Expression in C. Elegans Germ Cells. *Cell Rep.* **2012**, *2*, 1169–1177, doi:doi:10.1016/j.celrep.2012.09.019.
72. Liu, T.; Rechtsteiner, A.; Egelhofer, T.A.; Vielle, A.; Latorre, I.; Cheung, M.-S.; Ercan, S.; Ikegami, K.; Jensen, M.; Kolasinska-Zwierz, P.; et al. Broad Chromosomal Domains of Histone Modification Patterns in C. Elegans. *Genome Res* **2011**, *21*, 227–236, doi:10.1101/gr.115519.110.
73. Pauler, F.M.; Sloane, M.A.; Huang, R.; Regha, K.; Koerner, M.V.; Tamir, I.; Sommer, A.; Aszodi, A.; Jenuwein, T.; Barlow, D.P. H3K27me3 Forms BLOCs over Silent Genes and Intergenic Regions and Specifies a Histone Banding Pattern on a Mouse Autosomal Chromosome. *Genome Research* **2009**, *19*, 221–233, doi:10.1101/gr.080861.108.
74. Cai, Y.; Zhang, Y.; Loh, Y.P.; Tng, J.Q.; Lim, M.C.; Cao, Z.; Raju, A.; Lieberman Aiden, E.; Li, S.; Manikandan, L.; et al. H3K27me3-Rich Genomic Regions Can Function as Silencers to Repress Gene Expression via Chromatin Interactions. *Nature Communications* **2021**, *12*, doi:10.1038/s41467-021-20940-y.
